# IFITM3 Inhibits Influenza A Virus Infection by Preventing Cytosolic Entry

**DOI:** 10.1371/journal.ppat.1002337

**Published:** 2011-10-27

**Authors:** Eric M. Feeley, Jennifer S. Sims, Sinu P. John, Christopher R. Chin, Thomas Pertel, Li-Mei Chen, Gaurav D. Gaiha, Bethany J. Ryan, Ruben O. Donis, Stephen J. Elledge, Abraham L. Brass

**Affiliations:** 1 Ragon Institute of Massachusetts General Hospital, Massachusetts Institute of Technology and Harvard University, Charlestown, Massachusetts, United States of America; 2 Influenza Division, Centers for Disease Control and Prevention, Atlanta, Georgia, United States of America; 3 Department of Genetics, Harvard Medical School, Department of Genetics, Brigham and Women's Hospital, Boston, Massachusetts, United States of America; 4 Howard Hughes Medical Institute, Chevy Chase, Maryland, United States of America; 5 Gastrointestinal Unit, Department of Medicine, Massachusetts General Hospital, Boston, Massachusetts, United States of America; Washington University School of Medicine, United States of America

## Abstract

To replicate, viruses must gain access to the host cell's resources. Interferon (IFN) regulates the actions of a large complement of interferon effector genes (IEGs) that prevent viral replication. The interferon inducible transmembrane protein family members, IFITM1, 2 and 3, are IEGs required for inhibition of influenza A virus, dengue virus, and West Nile virus replication *in vitro*. Here we report that IFN prevents emergence of viral genomes from the endosomal pathway, and that IFITM3 is both necessary and sufficient for this function. Notably, viral pseudoparticles were inhibited from transferring their contents into the host cell cytosol by IFN, and IFITM3 was required and sufficient for this action. We further demonstrate that IFN expands Rab7 and LAMP1-containing structures, and that IFITM3 overexpression is sufficient for this phenotype. Moreover, IFITM3 partially resides in late endosomal and lysosomal structures, placing it in the path of invading viruses. Collectively our data are consistent with the prediction that viruses that fuse in the late endosomes or lysosomes are vulnerable to IFITM3's actions, while viruses that enter at the cell surface or in the early endosomes may avoid inhibition. Multiple viruses enter host cells through the late endocytic pathway, and many of these invaders are attenuated by IFN. Therefore these findings are likely to have significance for the intrinsic immune system's neutralization of a diverse array of threats.

## Author Summary

Influenza epidemics exact a great toll on world health. Thus research to identify new anti-influenza virus strategies would be useful. Each of our cells contains antiviral factors that work to inhibit infection. A large component of this antiviral program is regulated by the interferon family of signaling molecules. Here, we seek to better understand how one of these antiviral factors, IFITM3, contributes to both baseline, as well as interferon-induced, antagonism of influenza A viral infection. We found that interferon prevents influenza A virus from entering our cells by blocking the virus' fusion with the cellular membrane. Furthermore, we learned that IFITM3 is required for this antiviral action of interferon, and that high levels of IFITM3 alone can produce a similar viral inhibition. Together, these results improve our understanding of how IFITM3 serves to defend us against viral invasion at a very early stage of infection.

## Introduction

The 2009 H1N1 pandemic provided a strong reminder of the threat that influenza A virus poses to world health (http://www.cdc.gov/h1n1flu/cdcresponse.htm). The most effective means of protection against influenza is the seasonal vaccine. However, if the vaccine does not match the viral strains, its effectiveness can be reduced to 50% or less [1], [2]. Among small molecules, only two approved influenza drugs remain effective, zanamivir (Relenza) and oseltamivir (Tamiflu). Although resistance to zanamivir is rare, there has been an increase in oseltamivir-resistant flu strains [3]. Of concern, both drugs target viral neuraminidase (NA), precluding combinatorial therapy to minimize resistance [4], [5]. Thus, research to identify new anti-influenza strategies would be useful.

The influenza A virus is 50–100 nm in size, encodes for up to 11 proteins, and contains eight segments of negative single-stranded genomic RNA (3). Influenza A virus infection initiates with the cleavage and activation of the viral hemaglutinnin (HA) envelope receptor by host proteases [6], [7], [8], [9]. HA then binds to sialylated proteins on the cell surface, eliciting endocytosis of the viral particle. Endocytosed viruses are transported through the early and late endosomes, with late endosomal acidification triggering a conformational change in HA which results in viral-host membrane fusion [6], [10]. Fusion transitions from a hemifusion intermediate into a fusion pore through which the virus' eight viral ribonucleoproteins (vRNPs) enter the cytosol. The vRNPs are subsequently guided by the host cell's karyopherins into the nucleus [11], [12], [13], wherein the viral RNA-dependent RNA polymerase synthesizes viral genomes (vRNA) and mRNAs, both of which are exported to the cytosol, culminating in the production of viral progeny.

Genetic screens have identified multiple host factors and pathways which modulate influenza A virus infection *in vitro* [14], [15], [16], [17]. Using such a genetic screen, we identified the IFITM protein family members IFITM1, 2 and 3 as antiviral factors capable of blocking influenza A viruses [14]. We further tested the antiviral activity of IFITM3 protein using the seasonal influenza A strains, A/Uruguay/716/07 (H3N2) and A/Brisbane/59/07 (H1N1), and found similar levels of IFITM3-mediated viral inhibition [14]. IFITM3 accounts for a significant portion (50–80%) of IFN's (type I or II) ability to decrease influenza A virus infection *in vitro*, and IFITM3 resides in vesicular compartments that are IFN-inducible [14]. In addition, the IFITM family inhibits infection by the flaviviruses, dengue virus and West Nile virus [14], [18], as well as the filoviruses, Ebola and Marburg, and the SARS coronavirus [19]. The IFITM proteins also block vesicular stomatitis virus-G protein (VSV-G)-mediated entry, but do not substantially alter the replication of Moloney leukemia virus (MLV), several arena viruses, or hepatitis C virus (HCV, [14], [20]).

The human IFITM proteins were identified 26 years ago based on their expression after IFN stimulation [21], [22], [23]. The IFITM1, 2, 3 and 5 genes are clustered on chromosome 11, and all encode for proteins containing two transmembrane domains (TM1 and 2), separated by a conserved intracellular loop (CIL, [22]), with both termini extra-cellular or intra-vesicular [24], [25]. TM1 and the CIL are well conserved between the IFITM proteins and a large group of proteins representing the CD225 protein family. CD225 family members exist from bacteria (125 members) to man (13 members, with 156 members in chordata), with no in depth functional data available for any member other than the IFITM proteins. IFITM1, 2 and 3 are present across a wide range of species including amphibians, fish, fowl and mammals. The IFITM proteins have been described to have roles in immune cell signaling and adhesion, cancer, germ cell physiology, and bone mineralization [25], [26], [27], [28], [29], [30]. IFITM3 expression can inhibit the growth of some IFN-responsive cancer cells [31]. Genetic evidence also points to IFITM5/Bril being required for early bone mineralization [30], [32]. *Ifitm*Del mice, which are null for all five of the murine *Ifitm* genes, display a 30% perinatal mortality among null pups, but thereafter grow and develop normally in a controlled setting [26]. However, cells derived from these *Ifitm*Del mice are more susceptible to influenza A virus infection *in vitro* [14]. IFITM3 inhibited infection by all influenza A virus strains tested including a 1968 pandemic isolate and two contemporary seasonal vaccine viruses [14]. We have found IFITM3 to be the most potent of the IFITM protein family members in decreasing influenza A virus replication [14].

Viral pseudoparticles are differentially inhibited by the IFITM proteins based on the specific viral receptors expressed on their surfaces [14], [19]. Therefore, we have hypothesized that IFITM proteins inhibit susceptible virus families (Orthomyxoviridae, Flaviviridae, Rhabdoviridae, Filoviridae, and Coronaviridae) during the envelope-dependent early phase of the infection cycle, which extends from viral binding to cell surface receptors through the creation of the fusion pore between viral and host membranes [14], [19], [20]. In support of this notion, recent work demonstrated that IFITM protein overexpression did not prevent influenza A virions from accessing acidified compartments [19]. Consistent with its acting on endocytosed viruses, a portion of IFITM3 resides in structures that contain host cell endosomal and lysosomal proteins [19]. Furthermore, inhibition of influenza A virus infection depends on the palmitoylation of IFITM3, a post-translational modification that targets proteins to membranous compartments [33].

Here we directly test the idea that IFITM3 restricts influenza A viral infection during the envelope-dependent early phase of the viral lifecycle. Consistent with previous studies, we find that IFITM3 inhibits influenza A viral infection after viral-host binding and endocytosis, but prior to primary viral transcription [19], [20]. Moreover, using a combination of assays, we find that either IFN or high levels of IFITM3 impede influenza A viruses from transferring their contents into the host cell cytosol, and that IFITM3 is necessary for this IFN-mediated action. Therefore, we conclude that IFN is acting predominantly through IFITM3 to block viral fusion. We also find that IFN expands the late endosomal and lysosomal compartments, and that IFITM3 overexpression is sufficient for this phenotype. This study also presents data showing that IFITM3 overexpression leads to the expansion of enlarged acidified compartments consisting of lysosomes and autolysosomes. Interestingly, we observe that viruses trapped in the endocytic pathway of IFITM3-overexpressing cells are trafficked to these expanded acidified compartments. Based on these results and those of others [19], [20], we present a model whereby IFN acts via IFITM3 to prevent viral fusion, thereby directing endocytosed viruses to lysosomes and autolysosomes, for subsequent destruction. Collectively this study expands our understanding of how IFITM3 restricts a growing number of viruses by exploiting a shared viral vulnerability arising from their use of the host's endocytic pathway.

## Results

### IFITM3 inhibits influenza A viral infection after viral-host binding but prior to viral transcription

**Figure 1:**
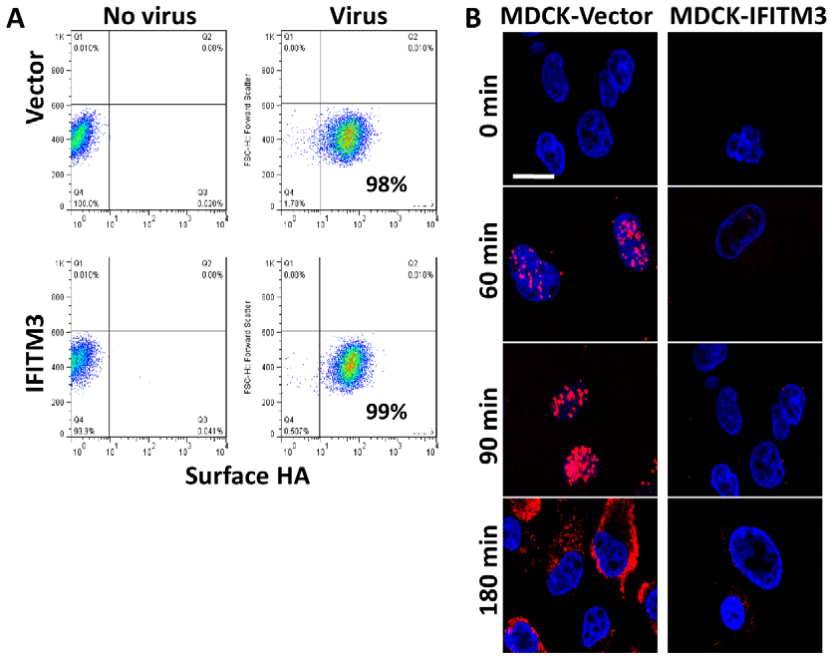
IFITM3 inhibits infection after viral binding but before viral transcription. A) A549 cell lines were incubated on ice with H1N1 WSN/33 to permit viral-host binding. Cells were washed, fixed and immunostained for surface-bound HA protein, then analyzed by flow cytometry. Values given are percentage of cells staining for surface HA. Values are representative of three independent experiments. B) MDCK cells transduced with the empty vector control (Vector) or IFITM3 were incubated with A/Puerto Rico/8/34 H1N1 (PR8) on ice. Warm media was added at time zero. Cells were then fixed at the indicated time points and hybridized with RNA probes against the viral NP mRNA (red) and stained for DNA (blue), then imaged by confocal microscopy. Images are representative of three independent experiments. (Scale bar: 20 µm).

The inhibition of HA-expressing pseudoparticles by the IFITM proteins pointed towards restriction occurring during the envelope-dependent phase of the viral lifecycle [14]. Therefore we tested IFITM3's impact on the most proximal phase of infection, viral binding, by incubating influenza A virus A/WSN/33 H1N1 (WSN/33, multiplicity of infection (moi) 50) with A549 lung carcinoma cells either stably overexpressing IFITM3 (A549-IFITM3) or an empty vector control cell line (A549-Vector, Fig. 1A). Samples were incubated on ice to permit viral binding but prevent endocytosis. After incubation, cells were washed with cold media, fixed and stained for HA. When analyzed by flow cytometry, we observed no appreciable difference in surface bound HA between the vector and IFITM3 cells. There was also no difference in surface-bound virus over a series of ten-fold dilutions of viral supernatant (data not shown). We also determined that the stable expression of IFITM3 did not alter the surface levels of (α2, 3) or (α2,6) sialylated cell-surface proteins (Fig. S1).

To investigate IFITM3's impact on initial viral mRNA production, we infected canine kidney cells, either expressing IFITM3 (MDCK-IFITM3) or the empty vector (MDCK-Vector), with influenza A virus (A/Puerto Rico/8/34 H1N1 (PR8), moi 500). We used PR8 because of the purified high titer stocks available. Next, the viral supernatant was removed and warm media was added (0 min). At the indicated times, cells were processed and stained for the positive stranded NP mRNA of PR8 using a specific RNA probe set (red, Fig. 1B), then imaged on a confocal microscope. Based on NP mRNA staining, primary viral transcription begins by 60 min. p.i. in the vector control, with the NP mRNA signal increasing through to 180 min., when the export of viral mRNAs to the cytosol can be observed. A decrease in primary viral transcription can be seen when comparing the IFITM3 cells to the vector control line. Therefore, IFITM3 inhibits influenza A viral infection after viral-host binding but before primary viral mRNA transcription.

### IFN interferes with vRNP nuclear entry and IFITM3 is necessary and sufficient for this antiviral defense

**Figure 2:**
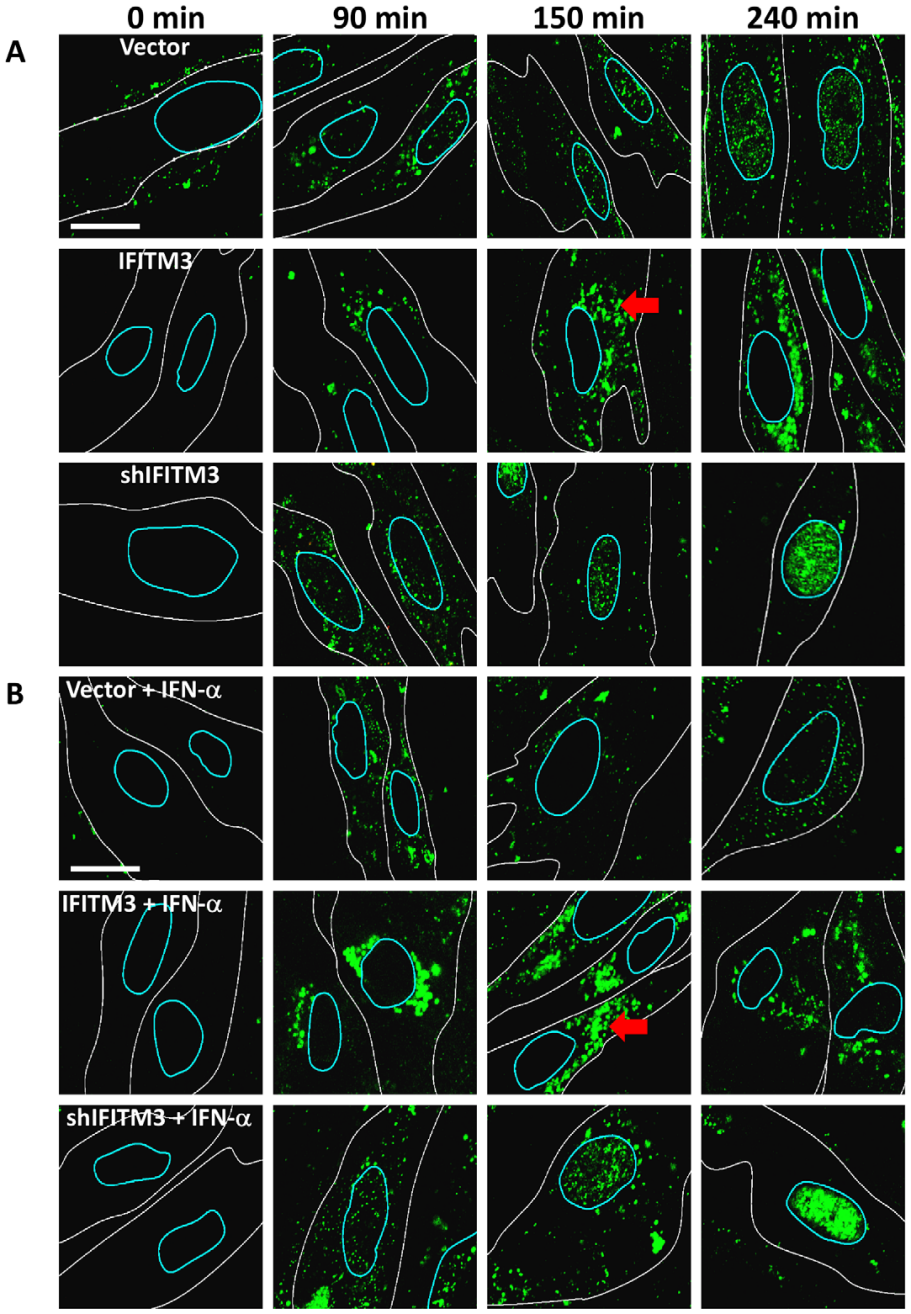
IFN prevents vRNP nuclear entry, and IFITM3 is necessary and sufficient for this action. A) Normal diploid human fibroblasts (WI-38 cells) were stably transduced with retroviruses containing either IFITM3 (IFITM3), a shRNA against IFITM3 (shIFITM3), an empty viral vector alone (Vector), or a non-targeting control shRNA (shScramble, Fig. S2). Cells were incubated with PR8 on ice, and then warm media was added at time zero. Cells were fixed at the indicated times p.i. and stained for NP (green) and DNA and analyzed by confocal microscopy. Image analysis software was used to define each cell's cytosolic (white lines) and nuclear peripheries (blue lines, based on DIC images and DNA staining, respectively). Red arrows: cytosolic compartments containing NP. Images are representative of four independent experiments. (Scale bar: 15 µm). B) As in (A) except that cells were treated with IFN-α prior to infection.

We next used confocal imaging to track the nuclear translocation of vRNPs (Fig. 2 [34], [35]). At the start of infection, the NP within infected cells is complexed with viral genomic RNA forming vRNPs. Therefore, immunostaining for NP permitted us to follow vRNP distribution intracellularly [16], [34], [36]. Normal diploid human lung fibroblasts (WI-38 cells) were stably transduced with empty vector (Vector), IFITM3 cDNA (IFITM3), or short hairpin RNAs (shRNA) either against IFITM3 (shIFITM3) or a scrambled non-targeting control (shScramble, Fig. 2, S2). WI-38s were chosen because of their normal karyotype and relatively larger and flatter morphology. Cells were first incubated on ice with PR8 (moi 500). Next, the viral supernatant was removed and warm media was added (0 min). At the indicated times after warming, cells were fixed, permeabilized, stained for NP and DNA, and imaged on a confocal microscope. Image analysis software was used to create an outline of each cell's periphery (white lines) and nucleus (blue lines). Based on NP staining, vRNPs arrive in the nuclei by 90 min in the vector control, shIFITM3, and in the shScramble cells, with the NP signal increasing through to 240 min (Fig. 2A, S2A–D).

In contrast, we observed decreased nuclear and increased cytosolic NP staining in the IFITM3 cells (Fig. 2, S2C). Moreover, in the IFITM3 cells greater than 60% of the cytosolic NP colocalized with Lysotracker Red (LTRed), a dye which marks acidic cellular compartments (late endosomes, lysosomes, pH≤5.5), and which was added to the warm media at time zero (Fig. S2A, D). The increased NP in the cytosol of the IFITM3 cells likely arises in part from an increase in the local concentration of viruses because α-NP Western blots (after trypsinizing the cells to remove adherent NP) did not show substantial differences in internalized NP levels between cell lines for up to 90 min post infection (p.i., data not shown). Because IFITM3 is required for the anti-viral actions of IFN *in vitro* [14], we performed a companion experiment with the WI-38 cells treated with IFN-α (Fig. 2B). IFN-α treatment also decreased NP nuclear staining in the WI-38-Vector cells, however this block was not as complete nor was it associated with similar levels of cytosolic NP staining as those seen with high levels of IFITM3. Consistent with the gain-of-function data, the depletion of IFITM3 decreased IFN's ability to block vRNP trafficking to the nucleus (Fig. 2A and B, compare top and bottom rows).

Similar results were obtained either using A549 cells (Fig. S3) or using MDCK cells, with the latter experiments employing additional influenza A viral strains (X:31, A/Aichi/68 (Aichi H3N2), Fig. S4A–C, WSN/33 and A/Victoria/3/75 H3N2, data not shown). It is important to note that the levels of IFITM3 protein in the A549-IFITM3 cells are higher than those seen after treatment with IFN-α or -γ (Fig. S3C). However, we have not observed that other overexpressed proteins have either protected against viral infection or expanded the lysosome/autolysosome compartment (data not shown), arguing that this is a specific effect. To better assess the expanded LTRed compartments observed with IFITM3 overexpression, we created MDCK cells stably expressing the lysosomal protein, LAMP1, fused to a red fluorescence protein (LAMP1-RFP) and IFITM3. As compared to control cells, the IFITM3 cells demonstrated extensive colocalization (>60%) between the NP and LAMP1-RFP signals, revealing that the entering viruses are trafficked to lysosomal compartments (Fig. S5).

**Figure 3 ppat-1002337-g003:**
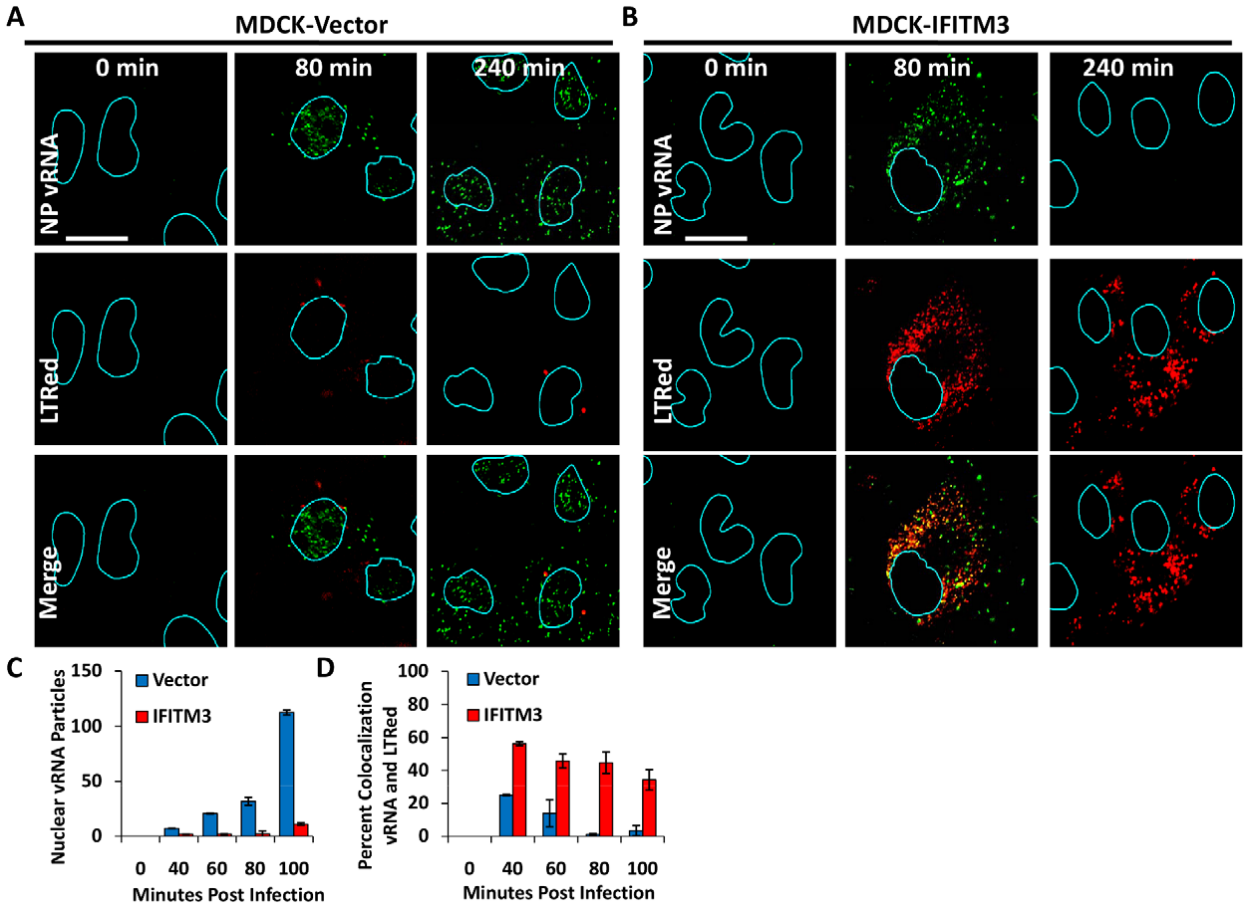
IFITM3 overexpression leads to both a retention of viral genomes in the cytosol, and a decrease in viral genomes entering the nucleus. MDCK cells transduced with the empty vector control (A) or IFITM3 (B) were incubated with PR8 on ice. Warm media containing lysotracker red dye (LTRed, red) was added at time zero. Cells were then fixed at the indicated time points and hybridized with RNA probes against the viral NP genome (NP vRNA, green) and stained for DNA, then imaged by confocal microscopy. Image analysis software was used to define the nuclear boundaries (blue lines) based on DNA staining. Images are representative of four independent experiments. (Scale bar: 20 µM). C) Quantitation of nuclear vRNA particles. The number of viral RNA particles per nucleus of the MDCK-Vector and IFITM3 cells at the indicated time points are shown. Values represent the mean +/− the SD of three independent experiments. D) Percent colocalization of vRNA and LTRed-containing compartments for MDCK-Vector and IFITM3 cells lines treated as in A and B, at the indicated time points.

We extended this analysis by directly tracking the location of the vRNA contained in the incoming vRNPs. MDCK cells stably expressing an empty vector or IFITM3, were used in time-course experiments as above (Fig. 3A–D). At the indicated times, cells were processed and stained for the negative stranded NP vRNA of PR8 using a specific RNA probe set (green). As seen with the WI-38 cells, we observed the nuclear translocation of vRNA by 80 min p.i. in the MDCK-vector cells (Fig. 3A). The nuclear vRNA signal was strongly decreased with IFITM3 overexpression based on the average number of vRNA particles present per nucleus (Fig. 3C). Consistent with the WI-38 results, the vRNAs accumulated in the cytosol of the IFITM3 cells, with >50% co-localizing with LTRed-staining acidic structures (Fig. 3D). Similar levels of retained cytosolic vRNPs were observed in experiments without LTRed (data not shown). Interestingly, we observed the loss of the vRNA signal in the acidic inclusions of the MDCK-IFITM3 cells between 80 and 240 min. p.i. (Fig. 3B). By comparison, the vRNAs in the control cells increased in number in both the nucleus and cytosol, as would be expected with the nuclear export of newly synthesized viral genomes [36].

**Figure 4:**
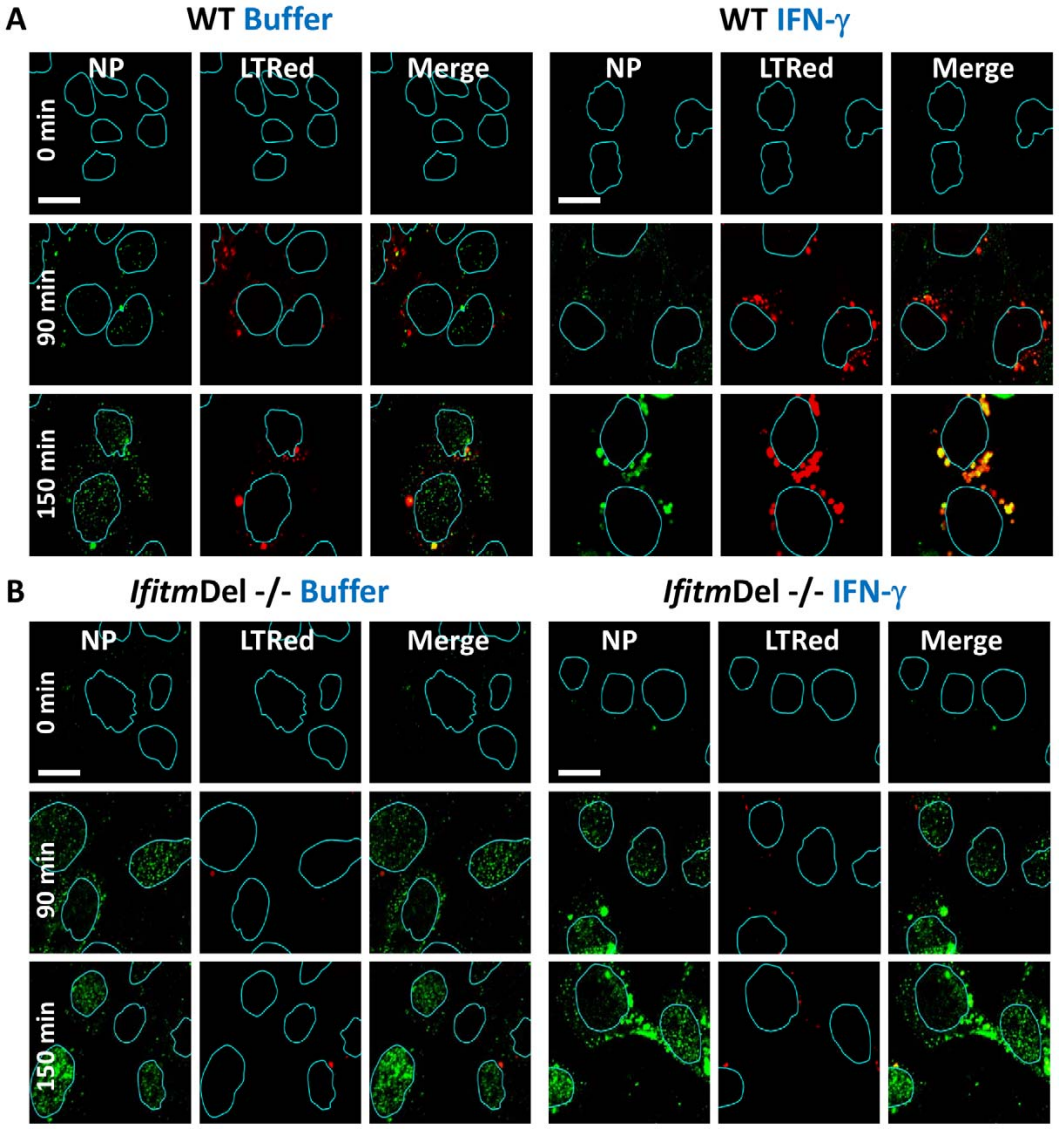
*Ifitm* knockout cells are more vulnerable to vRNP nuclear entry and are rescued by the restoration of Ifitm3 expression. MEFs, either A) wild type (WT) or B) *Ifitm*Del−/−, which are missing all five of the mouse *Ifitm* proteins, were either left untreated (left panels, Buffer), or treated (right panels) with IFN-γ. The following day cells were incubated with PR8 on ice. Cells were next incubated in warm media containing LTRed. Cells were then fixed at the indicated times and immunostained with anti-NP antibodies (green), stained for DNA (blue), and imaged by confocal microscopy. Image analysis software was used to define the nuclear boundaries (blue lines). Images are representative of three independent experiments. (Scale bar: 12 µm).

We next evaluated vRNP translocation in murine embryonic fibroblasts (MEFs) derived from animals that have had all five *Ifitm* genes deleted (*Ifitm*Del−/−, [14], [26]). Compared to wild-type (WT) matched litter mate controls, the *Ifitm*Del−/− MEFs displayed 5–10 fold more nuclear NP staining, with or without IFN-γ treatment (Fig. 4, S6C). IFN-mediated viral restriction was restored when we transduced the null MEFs with a retrovirus expressing Ifitm3 (*Ifitm*Del−/− *Ifitm3*, Fig. S6). Similar to what was observed with the IFITM3 overexpressing cell lines, the majority of the vRNP signal in the IFN-γ-treated WT and Ifitm3-rescued cells localized to acidic compartments (red, Fig. S6B). An increase in acidic compartments occurred after IFN-γ treatment with either the WT or the *Ifitm*Del−/−*Ifitm3* MEFs, but not in the *Ifitm*Del−/− cells, suggesting that Ifitm3 is required for this event (Fig. 4, S6). Similar results were obtained with IFN-α (data not shown). We conclude from these experiments using orthologous reagents (cell lines and influenza A viruses) and methods, that IFN impedes vRNP nuclear entry, and IFITM3 is necessary and sufficient for this activity.

### Viral pseudoparticle fusion mediated by either HA or VSV-G envelope proteins is decreased by IFN, and IFITM3 is necessary and sufficient for this activity

**Figure 5:**
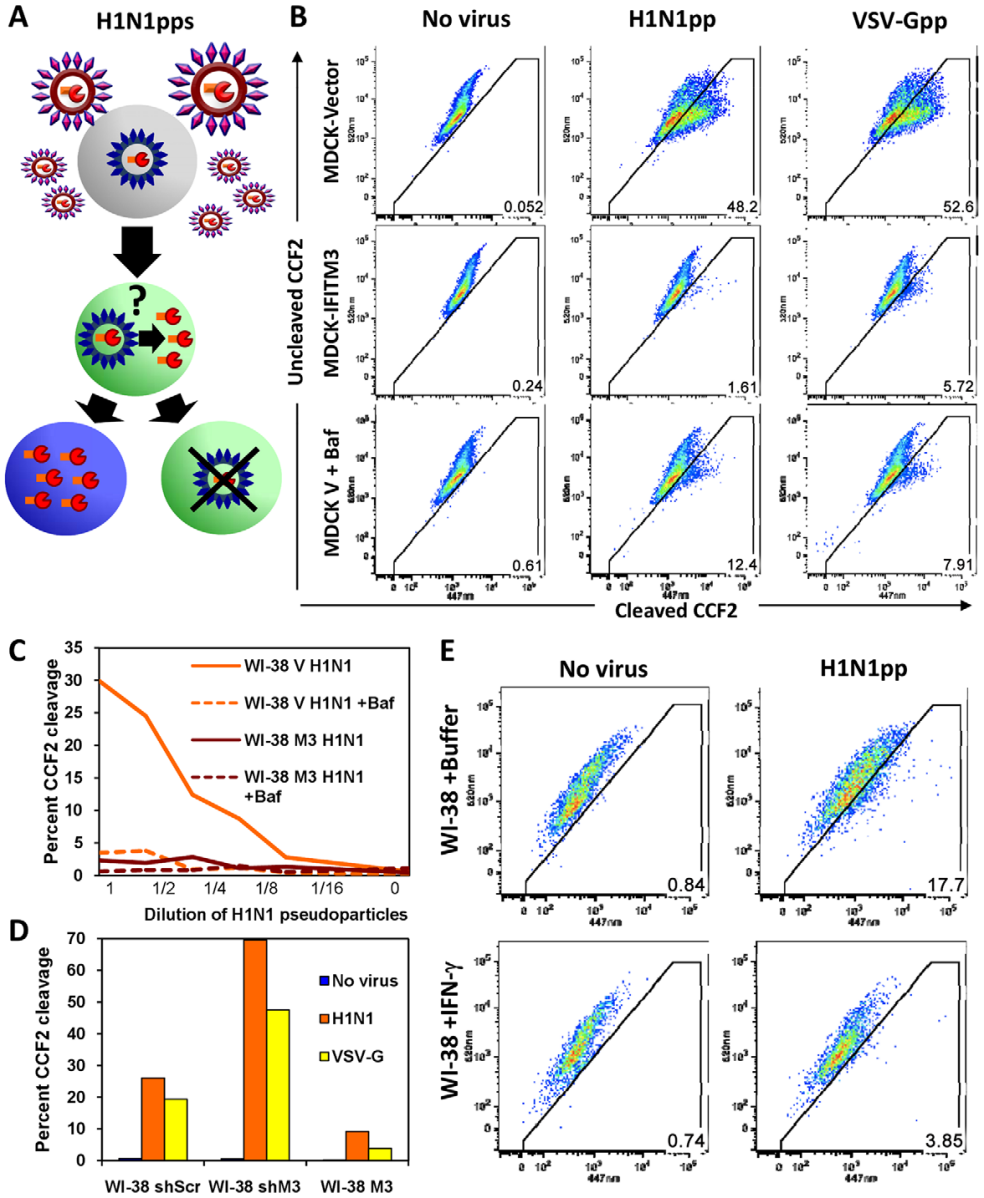
HA or VSV-G-mediated fusion is inhibited by IFN or IFITM3. A) Schematic model of the established viral fusion assay [37], [38] comprised of lentiviral pseudoparticles (pps) containing the β-lactamase protein fused to the HIV-1 accessory protein Vpr (BLAM-Vpr, shown in orange/red) and expressing HA and NA (WSN/33) on their surfaces. The H1N1pps were incubated for 2 h with cells, which were subsequently loaded with the β-lactamase flourogenic substrate, CCF2. Upon viral fusion, BLAM-Vpr enters the cytosol and can cleave CCF2, producing a wavelength shift from green to blue in emitted light when analyzed by flow cytometry ([37]). B) MDCK cells stably overexpressing IFITM3 (MDCK-IFITM3) or empty vector control cells (MDCK-Vector) were exposed for 2 h to viral pseudoparticles containing a BLAM-Vpr and expressing either the HA and NA envelope proteins of Influenza A virus (WSN/33, H1N1pp) or the VSV-G envelope protein (VSV-Gpp), then loaded with CCF2. After incubation with the indicated pseudoparticles, the cells were fixed and assayed for cleavage of CCF2 by determining the conversion of the fluorescence emission from 520 nm (uncleaved CCF2) to 447 nm (cleaved CCF2) using flow cytometry. Fusion of the pseudoparticles was inhibited by bafilomycin A1 (Baf). These results are representative of six independent experiments. C) IFITM3 inhibits fusion of H1N1pps in normal diploid fibroblasts. WI-38 fibroblasts stably transduced with IFITM3 (WI-38 M3) or the empty vector (WI-38 V) were exposed for 2 h to serial dilutions of H1N1pps containing BLAM-Vpr, with or without Baf. These results are representative of four independent experiments. D) Fusion of H1N1pps increases after IFITM3 knockdown. WI-38 fibroblasts stably transduced with a shRNA against IFITM3 (WI-38 shM3), a shRNA control with a scrambled sequence (WI-38 shScr), or the IFITM3 cDNA (WI-38 M3) were exposed to either no virus, H1N1pps or VSV-Gpps containing BLAM-Vpr. These results are representative of two independent experiments. E) Fusion of H1N1pps is inhibited by IFN-γ. WI-38 fibroblasts were treated with IFN-γ for 24 h or buffer alone prior to incubation with H1N1pps containing BLAM-Vpr. These results are representative of three independent experiments.

To further characterize the mechanism of IFITM3-mediated restriction, we used an established viral fusion assay [37], [38]. Lentiviral pseudoparticles containing the β-lactamase protein fused to the HIV-1 accessory protein Vpr (BLAM-Vpr) and expressing either HA and NA (H1N1, WSN/33), or VSV-G envelope proteins, were incubated for 2 h with cells, which were then loaded with the β-lactamase flourogenic substrate, CCF2. Upon viral pseudoparticle fusion, BLAM-Vpr enters the cytosol and cleaves CCF2, producing a wave length shift in emitted light (from green to blue) when analyzed by flow cytometry (Fig. 5A, [37]). In MDCK-IFITM3 cells we observed a decrease in both HA- and VSV-G-directed fusion, which was comparable to the block produced by poisoning of the host vacuolar ATPase (vATPases) with a low dose of bafilomycin A1 (Baf, Fig. 5B). The inhibition of vATPases prevents the low-pH activation required by these two viral envelope proteins to produce membrane fusion. A block to fusion of pseudoparticles expressing H1 (PR8), H3 (A/Udorn/72), H5 (A/Thai/74) or H7 (A/FPV/Rostock/34) subtypes of HA was also detected with MDCK cells or with chicken embryonic fibroblasts (ChEFs), in which IFITM3 strongly inhibited viral replication (Fig. S7A, B, C). In the case of the MDCK cells, the block to fusion closely paralleled the level of inhibition seen when the pseudoparticles were tested for productive infection using HIV-1 p24 expression as a readout (Fig. S7E). Consistent with earlier findings, pseudoparticles expressing an amphotropic MLV envelope protein were insensitive to IFITM3, showing the specificity of these results (Fig. S7D). Similarly to its effect on H5-expressing pseudoparticles, IFITM3 inhibited replication of infectious avian H5N1 influenza A virus, A/Vietnam/1203/04 (VN/04), isolated from a fatal human infection (Fig. S7F–H).

To enhance our analysis, we tested two additional cell lines, WI-38 and HeLa cells. A strong block to fusion in WI-38-IFITM3 cells, similar to that of the Baf and uninfected control samples, was seen at a range of serial dilutions of pseudoparticles, as well as an increase in fusion with IFITM3 depletion (shIFITM3, Fig. 5C, D). IFN treatment inhibited fusion of the H1N1 pseudoparticles, albeit to a lesser extent than IFITM3 overexpression (Fig. 5E), and this effect was largely absent when IFITM3 was stably depleted in HeLa cells (Fig. S8). Similar results were obtained with IFN-α (data not shown). Based on these experiments using multiple cell lines and HA, VSV-G, and MLV envelope-expressing pseudoparticles, we conclude that IFITM3 is required and sufficient for an IFN-mediated block of viral pseudoparticle fusion. Importantly, the increase in pseudoparticle fusion seen when endogenous IFITM3 was depleted in either the HeLa or WI-38 shIFITM3 cell lines argues that fusion inhibition underlies the first line defense provided by endogenous, as well as overexpressed, IFITM3.

MxA is an IFN-inducible large GTPase which interferes with secondary transcription during influenza A viral replication [39]. A549 cells express MxA and have been used extensively in influenza A viral replication studies [40]. Therefore to clarify the antiviral roles of IFITM3 and MxA, we tested the levels of viral replication in A549 cells stably expressing one of three shRNAs targeting IFITM3 (shIFITM3-1, -2, or -3). All three shIFITM3 cell lines showed increased infection (WSN/33 strain) and strong IFITM3 knockdown, when compared to the negative control cell line expressing a shRNA against firefly luciferase (shLuc), with or without IFN treatment (Fig. S9A, B). The majority of the protective effect of either IFN-α or γ was lost in the shIFITM3 cell lines. We next confirmed both the baseline levels, as well as the IFN-inducibility of MxA in the A549 cells (Fig. S9C). We also determined that MxA was both present and IFN-inducible in WI-38 normal fibroblasts, another cell line used in loss-of-function experiments in this work (Fig. S9D). Furthermore, IF studies of WI-38 cells showed that MxA is expressed in an IFN-inducible vesicular pattern and that these structures did not appreciably co-localize with vesicles containing IFITM3 (Fig. S9E, [39]). We conclude that MxA is expressed in the A549 and WI-38 cell lines, but cannot fully compensate for loss of the antiviral actions of IFITM3.

### IFITM3 is present in endosomes and lysosomes and these compartments are expanded with IFITM3 overexpression or IFN treatment

**Figure 6:**
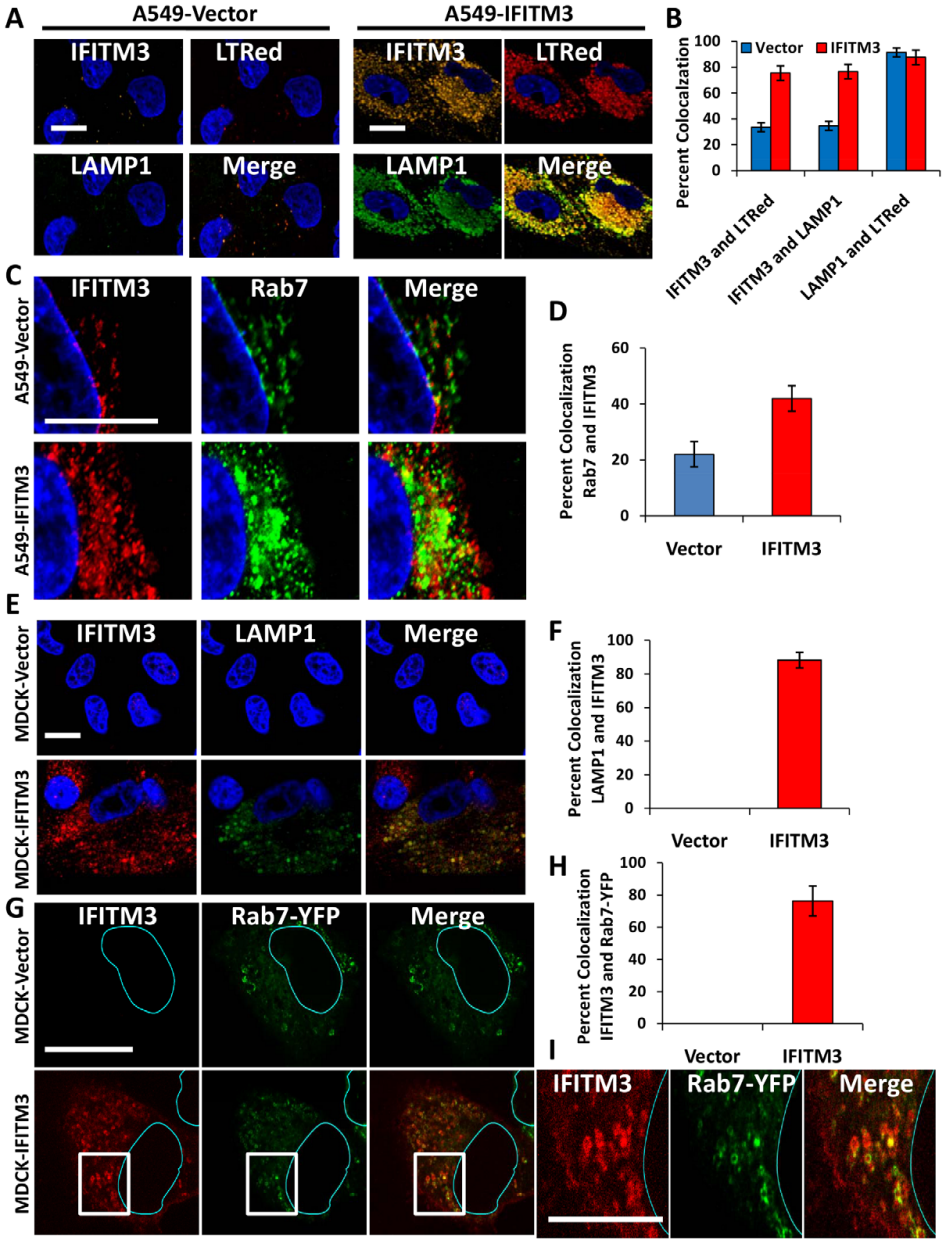
IFITM3 partially colocalizes with Rab7 and LAMP1, and compartments containing these proteins are amplified with IFITM3 overexpression. A) A549 cells stably transduced with either IFITM3 or with the empty vector alone, were incubated with LTRed (red) at 37°C, then fixed and immunostained for confocal imaging of IFITM3 (endogenous and overexpressed, gold), and LAMP1 (endogenous, green). DNA = blue. (Scale bars: 20 µM throughout). B) Percent colocalization of IFITM3, LTRed and LAMP in A549-Vector (blue) or IFITM3 (red) cells in (A). C) A549 cells stably transduced with either IFITM3 or with the empty vector alone were immunostained for confocal visualization of IFITM3 (endogenous and overexpressed, red) and Rab7 (endogenous, green). DNA = blue. D) Percent colocalization of IFITM3 and Rab7 in either the A549-Vector or A549-IFITM3 cells in (C). E) MDCK-Vector or MDCK-IFITM3 cells stained for exogenous IFITM3 (overexpressed, red) and LAMP1 (endogenous, green). DNA = blue. F) Percent colocalization of IFITM3 and LAMP1 in MDCK-Vector or MDCK-IFITM3 cells in (E). G) MDCK cells stably overexpressing Rab7-YFP and either IFITM3 (MDCK-IFITM3) or the empty vector control (MDCK-Vector) were immunostained and confocally imaged for IFITM3 (overexpressed, red) and Rab7-YFP (fluorescent signal from exogenous protein, green). Nuclear peripheries are represented by blue lines. H) Percent colocalization of Rab7-YFP and IFITM3 in either the MDCK-Vector or IFITM3 cells in (G). I) Enlarged view of images outlined by white boxes shown in (G), with MDCK-IFITM3 cells stably overexpressing both IFITM3 (red) and Rab7-YFP (green).

**Figure 7:**
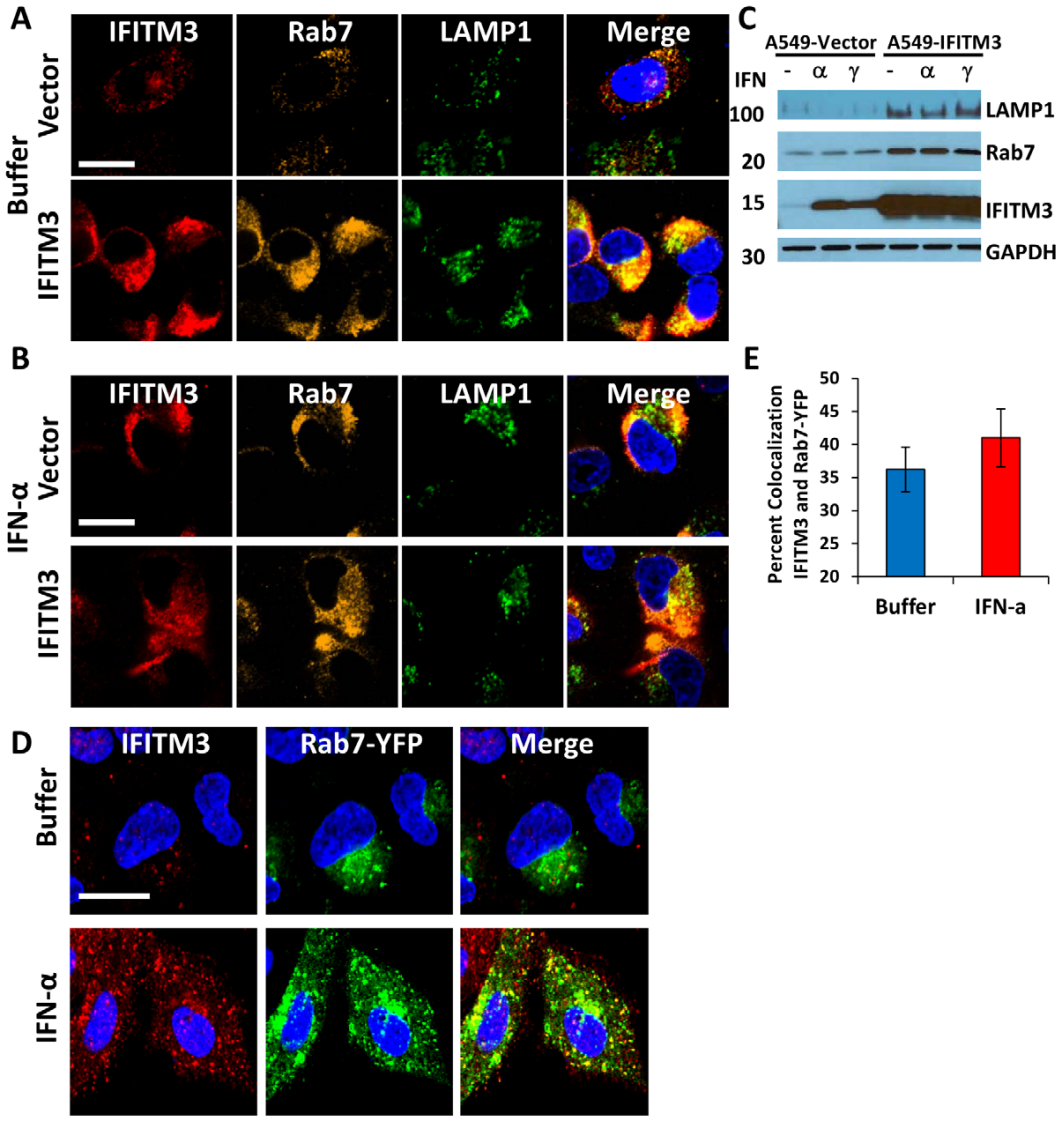
IFN treatment or IFITM3 overexpression expands late endosomes and lysosomes. A549 cells stably expressing IFITM3 (IFITM3) or empty vector (Vector) were (A) left untreated (Buffer) or (B) treated with IFN-α, then fixed, permeabilized and immunostained for IFITM3 (endogenous and overexpressed, red), Rab7 (endogenous, gold), LAMP1 (endogenous, green), and for DNA (blue, merged image). Images were obtained using a confocal microscope. Similar results were observed with IFN-γ (data not shown). (Scale bars: 20 µm throughout). C) Whole-cell lysates from A549-IFITM3 or A549-Vector cells in (A) and (B) treated or untreated with IFN-α or γ were subjected to immunoblotting against the proteins indicated. GAPDH levels are provided to demonstrate comparable protein loading. Molecular weights in kDa are provided to the left. These images are representative of three independent experiments. D) A549 cells stably expressing Rab7-YFP (fluorescent signal from exogenous protein, green) were left untreated (Buffer) or treated with IFN-α, then fixed, permeabilized and immunostained for IFITM3 (endogenous, red) and imaged confocally. DNA = Blue. Similar results were obtained for IFN-γ (data not shown). E) Percent colocalization of IFITM3 and Rab7-YFP in the A549 cells in (D), with or without IFN-α treatment.

Our data demonstrate that IFN or IFITM3 inhibit viral fusion. Influenza A virus fuses with the host membrane in late endosomes when the pH decreases to 5 [6], [7], [41]. Rab7 is a late endosomal/lysosomal small GTPase that is required for the fusion of many pH-dependent viruses, including influenza A virus [6], [41]. Previous reports have shown that IFITM3 colocalizes with LAMP1 and CD63, components of lysosomes and multivesicular bodies, respectively [19]. However, the relationship of IFITM3 and Rab7 within the host cell infrastructure remains unknown. Therefore we investigated the location of IFITM3, by undertaking immunoflourescence (IF) studies using antibodies that recognize IFITM3, Rab7, or LAMP1 [42]. Although the baseline level of IFITM3 in the A549-Vector cells was low, there was partial colocalization observed with either Rab7 or LAMP1 (Fig. 6A–D, 7A,). IFITM3 also partially colocalized with LAMP1 and LTRed-containing structures seen with IFITM3 overexpression (Fig. 6A, B, 7A). Interestingly, either IFITM3 overexpression or IFN increased the staining intensity of Rab7 and LAMP1 (Fig. 7A, B, S10A). Partial colocalization of IFITM3 was also seen with either endogenous LAMP1, or an exogenously expressed Rab7-yellow fluorescence fusion protein (Rab7-YFP) in MDCK cells (Fig. 6E–I). However, in all cases, co-localization was not complete because cells contained areas that uniquely labeled for each of the proteins. Western blots indicated that IFITM3 over-expression led to modest increases in both LAMP1 and Rab7 proteins in the A549-IFITM3 cells (Fig. 7C). However, these blots also showed that while IFN treatment of the A549-Vector cells increased IFITM3 protein levels as expected, the amount of Rab7 and LAMP1 remained unchanged. We conclude that IFITM3 partially resides in the late endosomal and lysosomal compartments along with Rab7 and LAMP1, and that IFITM3 overexpression or IFN treatment expands these compartments through a mechanism that cannot be fully explained by increased protein expression alone.

### IFITM3 overexpression leads to the expansion of the host cell's acidified compartments

**Figure 8:**
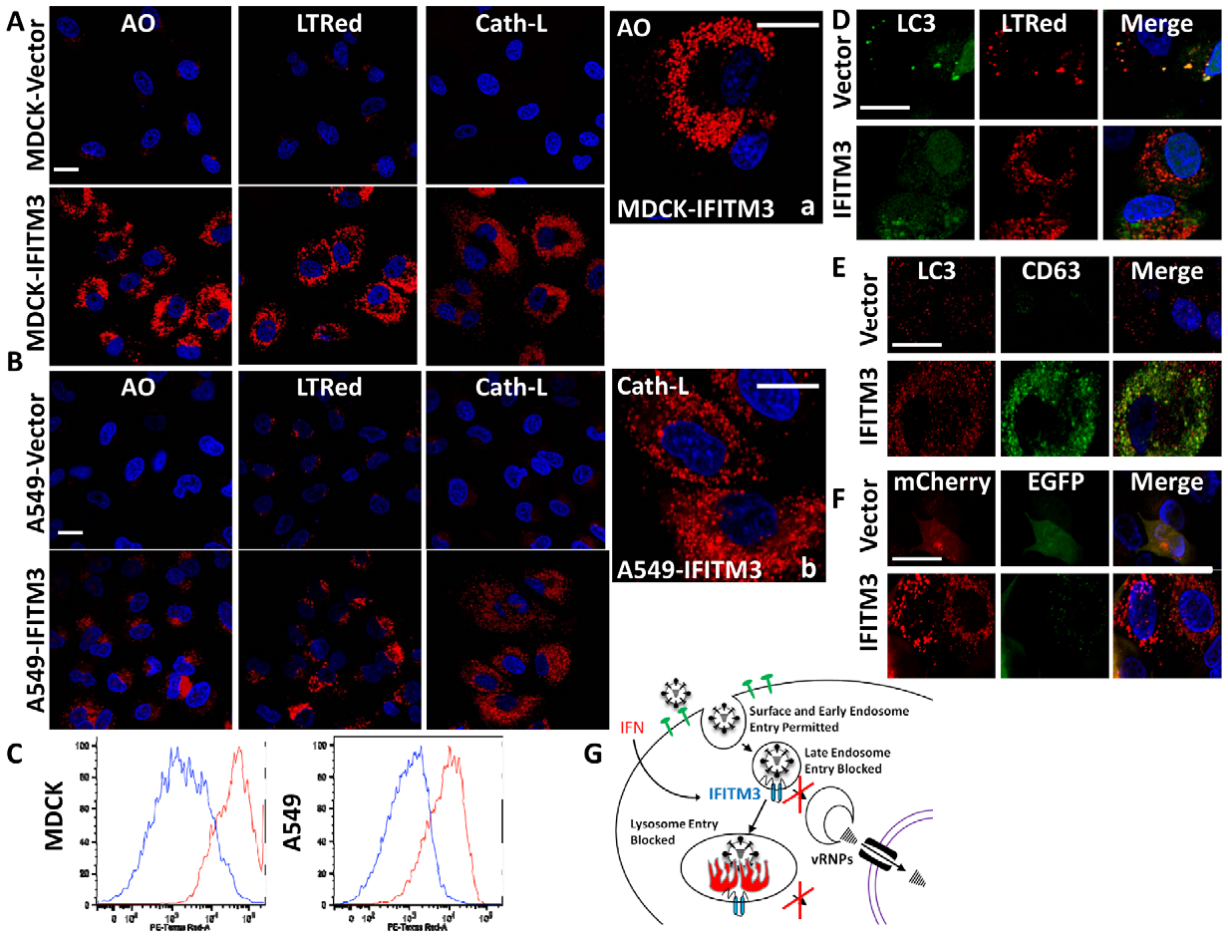
IFITM3 overexpression results in the expansion of acidified organelles. A) MDCK or (B) A549 cell lines, stably overexpressing IFITM3 or the empty vector alone, were incubated with either the acidophilic dye acridine orange (AO), LTRed, or a flourogenic cathepsin-L substrate (Cath-L). All cells were also stained for DNA (blue). After incubation cells were imaged on a confocal microscope. Middle panels show enlarged images of the IFITM3 cells. (Scale bars: 20 µm throughout). C) Vector (blue) or IFITM3 (red) transduced cell lines, either MDCK (left) or A549 (right), were incubated with LTRed then analyzed by flow cytometry. D) A549 cells stably transduced with IFITM3 or with the vector alone were incubated with LTRed (red), then immunostained for confocal imaging of LC3 (endogenous, green). DNA = blue. E) MDCK cells stably transduced with IFITM3 or with the vector alone were immunostained for confocal imaging of LC3 (endogenous, red) and CD63 (endogenous, green). DNA = blue. F) Confocal images of MDCK cells overexpressing IFITM3 or the empty vector alone showing the distribution and fluorescence intensities of a stably expressed mCherry-EGFP-LC3B fusion protein using fluorescence channels that detect light emitted from the mCherry protein, EGFP or both (merge). DNA = blue. G) Model of IFITM3-mediated restriction of virus replication. Endocytosed viruses enter late endosomes where IFITM3 is present. IFITM3 prevents viral fusion within the endosomes and likely lysosomes via an unknown mechanism, perhaps by altering pH, membrane characteristics, lipid composition, transport speed or destination. Trapped viruses are trafficked to lysosomes and/or autolysosomes where they undergo degradation.

Our assays showed that incoming influenza A viruses were retained in the expanded acidic compartments of both the IFITM3 overexpressing cell lines as well as the IFN-γ-treated MEFs, and that IFITM3 partially localized to these structures (Fig. 2–4, S2–4, S6). Therefore, we extended our investigation of these compartments. An increase in acidic structures was seen in MDCK and A549 cells overexpressing IFITM3 as compared to control cell lines, using either the vital acidophilic stain, acridine orange (AO), LTRed, or a cathepsin-L substrate that fluoresces only after it is proteolyzed, when compared to the corresponding vector control cells (Fig. 8A, B, a, b). Cathepsins are a family of lysosomal zymogens active in acidic environments (pH≤5.5) which are required for both the degradation of endocytic substrates and for the entry of several IFITM3-susceptible viruses [19]. Flow cytometry revealed an increase in the total LTRed fluorescent signal in both the MDCK and A549 IFITM3 cell lines when compared to controls (Fig. 8C). This expanded compartment represents a heterogeneous population of lysosomes and autolysosomes, based on confocal imaging showing the colocalization of the autophagosome marker, microtubule-associated protein 1 light chain 3 (LC3), with either LTRed or with CD63, with the latter being a resident of multivesicular bodies, amphisomes and autolysosomes (Fig. 8D, E). Furthermore, MDCK-IFITM3 cells stably transduced with an LC3 protein fused to both a red fluorescent protein (mCherry) and an enhanced green fluorescence protein (EGFP) showed a predominantly red signal, which occurs when the mCherry-EGFP-LC3 protein resides inside the acidified interior of an autolysosome (Fig. 8F, [43]). In keeping with previous reports that IFN-γ induces autophagy [44], [45], we detected enhanced LTRed staining in either IFN-γ treated MEFs or A549 cells (Fig. 4A, S10A). We conclude that increases in IFITM3 levels expand the lysosomal/autolysosomal compartment.

## Discussion

Here we report several novel findings regarding the antiviral actions of IFN and the transmembrane IEG, IFITM3. First, this study demonstrates that IFN inhibits the nuclear translocation of vRNPs, and that IFITM3 is required for this IFN-mediated block, with both endogenous and overexpressed IFITM3 inhibiting vRNP nuclear entry. Second, either endogenous or overexpressed IFITM3, as well as IFN treatment, block the fusion of viral pseudoparticles expressing various influenza A virus envelope proteins (H1, H3, H5 and H7 subtypes of HA), or the VSV-G envelope protein; this block is specific because the fusion of pseudoparticles expressing MLV envelope is not inhibited by IFITM3. Third, our work reveals that IFITM3 partially resides with Rab7 in late endosomes, thus placing it in position to block influenza A virus' cytosolic access. Fourth, IFITM3 overexpression or IFN induce the expansion of late endosomal and lysosomal compartments containing Rab7 and LAMP1. Fifth, we show that similar to IFN-γ treatment, IFITM3 overexpression expands the number and size of autolysosomes, and it is into these compartments that trapped viruses are trafficked and subsequently degraded. Consistent with previous reports, our data show that high levels of IFITM3 do not prevent viral access to acidified compartments and that IFITM3 colocalizes with CD63 and LAMP1 [19]. This is in contrast to a report noting the exclusion of overexpressed IFITM3 from LAMP1-containing structures [33]. Therefore, this work adds substantially to our interpretation of previous reports by demonstrating that key downstream events in the viral lifecycle, fusion and vRNP nuclear translocation, are prevented by either IFN or IFITM3. IFITM3 thus represents a previously unappreciated class of anti-viral effector that permits viral entry into the endosomal compartment, but prevents egress into the cytosol. These studies also raise new questions including i) how do IFN and IFITM3 prevent viral fusion? ii) how do IFN and IFITM3 alter the endosomal and autolysosomal compartments? and iii) is the latter action required for viral restriction, or alternatively does it arise as an outcome of IFITM3's potential cellular role?

Based on the substantial loss in IFN's potency observed when IFITM3 is depleted (50–80% loss of viral inhibition, Fig. S9A, B, [14]) we conclude that inhibition of viral emergence from the endosomal pathway is a prominent component of IFN's antagonism of influenza A virus replication *in vitro*. Our data also show that MxA cannot fully compensate for the loss of IFITM3 in IFN-treated cells challenged with influenza A virus. Recent work by Dittmann et al. [46] and Zimmermann et al. [47] reveal that human influenza A viral strains have evolved a means to evade MxA, suggesting a possible explanation for the cellular reliance on IFITM3 for protection *in vitro*. Similarly the IEG, IFIT1, prevents viral replication by targeting viral 5′ triphosphate-RNAs (PPP-RNA) for destruction [48], [49]. Given that IFITM3 is necessary for the majority of IFN-mediated restriction of influenza A virus *in vitro*, it may be that the virus has also evolved a means to at least partially nullify IFIT1, perhaps via the massive production of short “decoy” PPP-RNAs, as previously postulated [49], [50].

IFITM3 primarily resides in the endosomal compartment and partly colocalizes with Rab7 and LAMP1. IFITM3 overexpression or IFN stimulation caused the endocytosed viruses to accumulate in acidic compartments that contained both IFITM3 and LAMP1. Together with the BLAM-Vpr fusion assay data, these results reveal that IFITM3 prevents viral-host membrane fusion within late endosomes, and likely within lysosomes as well, in light of studies showing IFITM-mediated restriction of filoviruses and coronaviruses, which depend on cathepsin-mediated activation prior to fusion [19]. In doing so, IFITM3 traps the virus on a path which terminates in a degradative environment [51]. In support of this, our experiments show the eventual loss of a detectable vRNA signal in the LTRed-positive compartments of the IFITM3-transduced cells, thus revealing the fate of viral fitness under those conditions.

These studies also reveal that elevated levels of IFITM3 correlate with the expansion of host cell structures containing Rab7 and LAMP1, and that IFITM3 was also present in these structures. In the MEF and A549 experiments, IFN produced increased Rab7 and LAMP1 immunostaining, in addition to an increase in acidic structures. At present, we cannot explain the increased Rab7 and LAMP1 signals seen after IFN stimulation or IFITM3 overexpression solely on the slight elevations in the abundance of these proteins detected by immunoblotting. Two possible explanations for the increased immunostaining observed, are that IFN stimulation induced these proteins to cluster together or alternatively unmasked sequestered epitopes; we find the latter possibility less likely since LAMP1 and Rab7 flourescent fusion proteins also showed larger and more intense signals under similar conditions. We envision that IFITM3-mediated clustering of organelles and their protein cargoes might contribute to the host cell's antiviral state. Earlier work reported no correlation between the size of the IFITM3-induced acidified compartments and the level of viral restriction [19], however, we observe that increasing levels of IFITM3 result in both an expansion of lysosomes/autolysosomes and increased viral inhibition. These observations might be explained by a common mechanism underlying the increase in these structures and viral inhibition, in addition to raising the possibility that they play a role in IFITM-mediated viral restriction.

Is there a common characteristic shared by IFITM3-susceptible viruses? The late endosomal- and lysosomal-associated small GTPase, Rab7, is required for influenza A virus infection [7], [41]. The IFITM3-resistant viruses previously tested (MLV, the arena viruses and the hepacivirus, HCV) are all Rab7-independent, while the entry of the IFITM3-susceptible viruses (influenza A, dengue, Ebola, Marburg, and SARS) relies on Rab7 [14], [19], [41], [52], [53], [54]. Standing against this hypothesis, is the lack of effect on VSV-G-mediated entry with expression of a dominant negative Rab7 [41], [55], [56]). However, additional studies have shown that VSV-G-directed entry is dependent on transport to the late endosomes [57], [58]; these latter results, together with those of Huang et al. and Weidner et al. [19], [20], are consistent with the prediction that viruses that fuse in late endosomes or lysosomes are vulnerable to IFITM3's actions, while viruses whose genomes enter at the cell surface or in the early endosomes may avoid IFITM3's full effect. Of note, we have been unable to demonstrate that IFITM3 blocks HIV-1 replication using TZM-bl HeLa cells and are working to address these differences with a published study ([59], data not shown).

This study, together with previous work, demonstrates that IFITM3 permits endocytosis of viruses, but prevents viral fusion and the subsequent entry of viral contents into the cytosol [19], [20]. While the BLAM-Vpr fusion assay demonstrates inhibition of fusion by IFN or by IFITM3, we note that this assay uses an indirect readout to assess entry of viral contents. Therefore several possibilities could explain the containment and neutralization of viruses within the endosomal pathway, including alterations in endosomal trafficking, acidification, or the host membrane's fusion characteristics (bending modulus, elasticity). While additional work is required to further define the mechanism, the lack of toxicity seen with cells stably overexpressing high levels of IFITM3 suggests that gross alterations in endogenous trafficking or pH control are unlikely (data not shown). Therefore overexpressing or activating IFITM3 to produce an enhanced antiviral state may be an effective prevention strategy during high risk periods in vulnerable populations.

We propose that IFN causes the degradation of endocytosed viruses by preventing their contents from entering the host cytosol, and that IFITM3 is necessary and sufficient for this defense (Figure 8G). IFITM3's mode of defense could be envisioned as an effective means to neutralize pathogens during an organism-wide threat. Such actions might confer an advantage to the host because if IFITM3 simply decreased viral attachment and/or entry, the repulsed viruses would be free to attack neighboring cells. Of course while there are considerable differences between this simple scenario and the directed phagocytosis of pathogens by specialized immune cells, i.e. macrophages, the similarities none-the-less suggest an early prototype for a more evolved defense mechanism.

## Materials and Methods

### Cell lines and culture conditions

U2OS, A549, MDCK, HeLa cells (all from ATCC), and chicken embryonic fibroblasts (ChEFs, from Charles River Labs) were grown in complete media (DMEM, Invitrogen Cat#11965) with 10% FBS (Invitrogen). WI-38 cells (ATCC) were cultured in DMEM (Invitrogen Cat#10569), containing non-essential amino acids (Invitrogen Cat#11140) and 15% FBS. Wild type and matched *Ifitm*Del−/− MEFs were from adult *Ifitm*Del+/− mice [26] that were intercrossed and MEFs derived from embryos at day 13.5 of gestation, as described previously [14]. The MEFs were genotyped by PCR and Western blot, and the generation of the *Ifitm*Del−/− *Ifitm3* cells have been previously described [14].

### Plasmids

The IFITM3 retroviral vector, pQCXIP-IFITM3 and empty vector control (Clontech) have been previously described [14]. The shRNA lentiviral vectors, pLK0.1-Scramble and pLK0.1-shIFITM3-3 (clone ID HsSH00196729) are available from the Dana Farber DNA core, Harvard Medical School, Boston, MA. pCAGGS-HA WSN/33 and pCAGGS-NA WSN/33 were kind gifts of Dr. Donna M. Tscherne and Dr. Adolpho Garcia-Sastre, Microbiology Dept., Mt. Sinai School of Medicine, NY, NY [38]. pBABE-mCherry-EGFP-LC3B was from Addgene (Plasmid #22418) and was kindly deposited by Jayanta Debnath. pLZS-Rab7-YFP and pLVX-RFP-LAMP1 were generously provided by Walther Mothes, Section of Microbial Pathogenesis, Yale University School of Medicine. The following shRNA sequences (sense strand sequence provided) were cloned into the pAPM shRNA-expression lentiviral vector [60], to create the viruses used to generate the A549 IFITM3 knockdown cell lines in Fig. S9:

IFITM3-1: 5′-TCCTCATGACCATTCTGCTCAT-3′

IFITM3-2: 5′-CCCACGTACTCCAACTTCCATT-3′

IFITM3-3: 5′-TTTCTACAATGGCATTCAATAA-3′

### Viral propagation and titration

Influenza A virus A/Puerto Rico/8/1934 (H1N1) (PR8, Charles River Labs) and A/WSN/33 (H1N1) (kind gift of Dr. Peter Palese, Microbiology Dept., Mt. Sinai School of Medicine, NY, NY) were propagated and assessed for viral infectivity as previously described [14]. Influenza A virus A/Vietnam/1203/2004 (H5N1) was propagated and characterized as previously described [61].

### Cytokines

Human interferon (IFN)-γ (Invitrogen) was used at 100–300 ng/ml, human IFN-αA2 (PBL Interferon Source) was used at 500–2500 U/ml. Cells were incubated with cytokines for 16–24 h prior to IF or viral infection experiments unless otherwise noted. Murine IFN-γ (PBL Interferon Source) was used at 100–300 ng/ml.

### Western analysis

Whole-cell extracts were prepared by cell lysis, equivalent protein content boiled in SDS sample buffer, resolved by SDS/PAGE, transferred to Immobilon–P membrane (Millipore), and probed with the indicated antibodies.

### Time course infection experiments and confocal microscopy

Cells were seeded on glass coverslips for Influenza A virus infection experiments. Cells were incubated on ice with PR8 for 40 min. At time zero, the viral supernatant was removed and 37°C media was added with or without Lysotracker Red DND-99 (Invitrogen). At the indicated time points post-warming, cells were washed twice with D-PBS (Sigma) and incubated for 30 seconds with room temperature 0.25% trypsin (Invitrogen). The cells were then washed with complete media twice and fixed with 4% formalin (PFA, Sigma) in D-PBS. Image analysis for quantitation of vRNP nuclear translocation was done using Imaris 7.1 (bitplane scientific software). We generated a mask of the nucleus and applied this mask to the channel containing the viral signal (puncta) to determine vRNA puncta contained in each nucleus.

### Live cell imaging experiments

Cells were incubated at 37°C and 5% CO_2_ for 60 min. with either Lysotracker Red DND-99 or acridine orange (ImmunoChemistry Technologies). Hoechst 33342 (DNA stain, Invitrogen) was incubated (1∶10,000) with the cells for the final 15 min. The Cathepsin L flourogenic substrate assay was performed as per the manufacturer's instructions (Cathepsin L -Magic Red, ImmunoChemistry Technologies). Cells were visualized live by confocal microscopy.

### Immunoflourescence protein

Cells were fixed in 4% PFA in D-PBS, and then incubated sequentially in 0.25% Tween 20 (Sigma), then 1% BSA with 0.3 M glycine (Sigma), both in D-PBS. Primary and secondary antibodies are listed below. Slides were mounted in Vectashield with DAPI counterstain (Vector Labs). Slides were imaged using a Zeiss LSM 510, laser scanning inverted confocal microscope equipped with the following objectives: 40× Zeiss C-APOCHROMAT UV-Vis-IR water, 1.2NA, 63× Zeiss Plan-APOCHROMAT DIC oil, 1.4NA, and 100× Zeiss Plan-APOCHROMAT DIC oil, 1.46NA. Image analysis was performed using ZEN software (Zeiss). Laser intensity and detector sensitivity settings remained constant for all image acquisitions within a respective experiment. Nuclear outlines were generated using Metamorph software suite (Molecular Devices) using the Kirsch/Prewitt filter to define boundaries and then subtracting out the original binary images.

### Antibodies

The following antibodies were used in this study for either Western blotting (WB) or immunoflourescence (IF), or both as indicated, along with their respective source and catalogue number: Primary antibodies: Actin (Sigma A5316, WB), CD63 (Developmental Studies Hybridoma Bank (DSHB) clone H5C6, IF), Fragilis (mouse Ifitm3) (Abcam ab15592, WB, IF), GAPDH (BD Biosciences 610340, WB), HA (Wistar collection, Coriell Institute, clone H18-S210, WC00029, IF), IFITM3 (Abgent AP1153a, WB, IF), IFITM3 (Abgent AP1153c, IF), LAMP1 ((DSHB) clone H4A3, WB, IF), LC3 (Nanotools Mab LC3-5F10, WB, IF), MX1 (Proteintech 13750-1-AP, WB, IF), NP (Millipore clone H16-L10-4R5 MAB8800, IF), RAB7 (Abcam 50533, WB, IF). Secondary antibodies for IF (all from Invitrogen): Alexa Fluor 488 and 647 (goat anti-rabbit and goat anti-mouse). The LAMP1 [H4A3] and CD63 [H5C6] antibodies were developed by J.T. August and J.E.K. Hildreth and were obtained from the DSHB and developed under the auspices of the NICHD and maintained by The University of Iowa, Department of Biology, Iowa City, IA.

### Immunoflourescence RNA

These experiments employ the QuantiGene ViewRNA slide-based assay kit from Affymetrix (Cat #QV0096) with all components from that source unless noted. RNA was visualized following a modified manufacturer protocol; changes made include the omission of the ethanol dehydration step, and use of Vectashield mounting media. Post-fixation with 4% PFA, cells adherent on coverslips were incubated with 1× detergent solution or incubated in 0.25% PBS-Tween20. Cells were then incubated with Proteinase K. Next cells were incubated at 40°C in hybridization solution A containing a viewRNA probe set designed against either the negative stranded RNA NP genome (vRNA) of PR8 (Affymetrix VX1-99999-01 QG ViewRNA TYPE 1 Probe Set against NP Influenza A virus (A/PuertoRico/8/34(H1N1)) at 1∶100) or a probe set against the positive stranded NP mRNA. Cells were then incubated in hybridization preamplifiers (1∶100 in hybridization buffer B) at 40°C. Finally cells were incubated with labeled probes (1∶100 in hybridization buffer C), washed and imaged as above. All steps were followed by two D-PBS washes.

### BLAM-Vpr pseudoparticle fusion assays

Pseudotyped lentiviral particles expressing the HA envelope were produced by plasmid transfection of HEK 293T cells with an HIV-1 genome plasmid derived from pBR43IeG-nef+ (NIH AIDS Research and Reference Reagent Program (Division of AIDS, NIAID, NIH, Cat#11349, from Dr. Frank Kirchhoff) modified with a deletion which abolishes expression of Env without disrupting the Rev-responsive element, pCAGGS-HA WSN/33, pCAGGS-NA WSN/33 and pMM310, which encodes a hybrid protein consisting of β-lactamase fused to the HIV accessory protein, Vpr (NIH AIDS Research and Reference Reagent Program, Division of AIDS, NIAID, NIH (Cat#11444) from Dr. Michael Miller). pCG-VSV-G together with pBR43IeG-nef+ and pMM310 were transfected to produce VSV-G pseudotyped lentiviral particles. For the H5N1, H3N1, and H7N1 pseudoparticles, pCAGGS-HA5 (A/Thailand2(SP-33)/2004) pCAGGS-HA3 (A/Udorn/72), and pCAGGS-HA7 (A/FPV/Rostock/34) expression plasmids were co-transfected with the pCAGGS-NA WSN/33, pMM310, and the pBR43IeG-nef+ lentiviral backbone. Cultures for pseudoparticle fusion assays, including stably transduced MDCK cells and WI-38 fibroblasts, were plated in 24-well dishes with 90,000 cells per well at the beginning of each assay. At the time of assay, 0.5 mL of virus stock was added to cells and incubated for 2–3 h (depending on cell type) at 37°C. In experiments using bafilomycin A1 (Sigma), the inhibitor was added at 0.1 nM final concentration (low dose) at 37°C for 1 h prior to incubation with virus. After infection, viral media was then aspirated and replaced with complete DMEM containing CCF2-AM (Invitrogen) along with 1.7 µg/mL probenecid (Sigma). Cells were incubated in the dark for 1 h, followed by dissociation from the dish using Enzyme Free PBS-based Dissociation Buffer, and fixation in 2% PFA. Flow cytometry was conducted on a Becton Dickinson LSRII using 405 nm excitation from the violet laser, and measuring 450 nm emission in the Pacific Blue channel and 520 nm emission in the Pacific Orange channel. Data was analyzed using FACSDiva and FlowJo8.8.7.

### Sialic acid linkage expression studies

A549 cells stably transduced to overexpress IFITM3 or with empty expression vector (pQCXIP, Clontech) were grown to ∼50% confluency, dissociated with trypsin-free EDTA-based dissociation buffer (Invitrogen) for 10 min. at 37°C. Cells were incubated at 4°C with FITC-conjugated *Sambucus nigra* lectin (SNA, Vector Labs #FL-1301) to detect (α-2,6) sialic acid linkages, and biotinylated *Maackia amurensis* lectin II (MAL, Vector Labs #B-1265) to detect (α-2,3) sialic acid linkages, followed by streptavidin-PE-Cy7 (Invitrogen). Cells were incubated with lectins individually and in combination, and the results of staining were indistinguishable. All cells were stained with violet cell-impermeable dye (Invitrogen #L34955), and cells were included in the analysis if viable by FSC/SSC and viability dye.

### Binding assay

A549 cells transduced with IFITM3 or the empty vector pQXCIP were detached using Enzyme Free PBS-based Dissociation Buffer, and then washed in cold PBS extensively. Cells and virus (WSN/33) were pre-chilled on ice for 30 min. and mixed at a moi of 50 and incubated at 4°C for 1 h with rotation. Cells were washed extensively with ice cold PBS and then fixed using 4% PFA. The cells were then probed with anti-HA mouse monoclonal antibody (Wistar collection, Coriell Institute, clone H18-S210, WC00029, IF) for 1 h at room temperature, followed by anti-mouse AlexaFlour-488 conjugated antibody (Invitrogen) for 1 h with PBS washes in between, then analyzed by flow cytometry.

## Supporting Information

Figure S1 **IFITM3 overexpression does not alter the surface levels of (α-2,3) or (α-2,6) sialylated proteins.** A549 cells stably transduced with IFITM3 or the empty vector were incubated with biotinylated *Maackia Amurensis* lectin II (MAL) to detect (α-2,3) sialic acid linkages, followed by streptavidin-PE-Cy7, as well as FITC-conjugated *Sambucus Nigra* lectin (SNA) to detect (α-2,6) sialic acid linkages. A) The percentage of IFITM3 or vector cells staining positive for both sialic acid linkages (upper right hand quadrant), compared to unstained controls. B) IFITM3 overexpressing and vector cells are compared with regard to each sialic acid linkage in the double-stained populations.
(PDF)

Figure S2 **IFITM3 arrests influenza A virus in acidic cytosolic inclusions preventing vRNP nuclear translocation.** A) Normal diploid human fibroblasts (WI-38 cells) were stably transduced with retroviruses containing IFITM3 (WI-38 IFITM3) or (B) a non-targeting control shRNA (WI-38 shScramble). Cells were incubated with PR8 on ice, and then warm media containing LTRed (red) was added at time zero. Cells were fixed at 150 min. p.i. and stained for NP (green) and DNA, then analyzed by confocal microscopy. Image analysis software was used to define each cell's cytosolic (white lines) and nuclear peripheries (blue lines, based on DIC images and DNA staining, respectively). Images are representative of four independent experiments. (Scale bar: 12 µM). C) Quantitation of nuclear vRNP particles. The number of vRNP particles per nucleus of the WI-38 cell lines (with or without IFN treatment) at the indicated time points are shown. Values represent the mean +/− the SD of three independent experiments. D) Percent colocalization of vRNPs and LTRed compartments in WI-38 shScramble, shIFITM3 or IFITM3 expressing cells at the indicated times p.i. Values represent the mean +/− the SD of three independent experiments. E) Western blot of lysates from WI-38 cells probed with the indicated antibodies. shIFITM3-3 is referred to as shIFITM3 in the preceding figures and was selected for use based on its superior knockdown of the target protein.
(PDF)

Figure S3 **A549 cells overexpressing IFITM3 inhibit vRNP nuclear entry.** A549 cells overexpressing the empty vector control (A) or IFITM3 (B) were incubated with PR8 on ice (moi 500). At time zero warm media was added along with LTRed (red). At the indicated times, cells were processed and stained for NP (green) and DNA (blue lines represent the nuclear periphery based on staining), then imaged using a confocal microscope. (Scale bar: 20 µM). These images are representative of three independent experiments. C) Whole cell lysates of A549 cells used in (A) and (B) treated with either buffer, IFN-α or IFN-γ, were subjected to immunoblotting using the indicated antibodies.
(PDF)

Figure S4 **IFITM3 overexpression halts H3N2 influenza A virus in acidic cytosolic inclusions prior to vRNP nuclear translocation.** MDCK cells stably expressing (A) the empty vector control or (B) IFITM3 were incubated with Aichi H3N2 virus on ice, and then warm media was added at time zero along with LTRed (red). Cells were then fixed at the indicated times p.i. and stained for NP (green), and DNA (blue lines denote nuclear periphery), then imaged by confocal microscopy. Images are representative of three independent experiments. (Scale bar: 20 µm). B) Quantitation of nuclear vRNP particles. The number of vRNP particles per nucleus of the MDCK cell lines at the indicated time points are shown. Values represent the mean +/− the SD of three independent experiments. C) Percent colocalization of vRNP and LTRed compartments in MDCK-Vector and MDCK-IFITM3 cell lines at the indicated times p.i.
(PDF)

Figure S5 **vRNPs are retained in LAMP1-containing organelles in cells overexpressing IFITM3.** A) MDCK-Vector or IFITM3 cells stably expressing a LAMP1-red fluorescence protein (LAMP1-RFP) were challenged with PR8 as in Fig. S4. Cells were immunostained for NP (green), stained for DNA, and then imaged confocally along with the collection of LAMP1-RFP fluorescence (orange). Images are representative of three independent experiments. Blue lines represent the nuclear margins based on DNA staining. (Scale bar: 20 µM). B) Quantitation of nuclear vRNP particles. The number of vRNP particles present per nucleus of the MDCK cell lines at the indicated time points are shown. Values represent the mean +/− the SD of three independent experiments. C) Percent colocalization of vRNP particles and LAMP1-RFP-containing compartments in MDCK-Vector and MDCK-IFITM3 cell lines at the indicated times p.i. Values represent the mean +/− the SD of three independent experiments.
(PDF)

Figure S6 **Ifitm3 expression rescues IFN-γ-mediated inhibition of vRNP nuclear translocation in** ***Ifitm*** **Del** ***−/−*** **MEFs.** A) *Ifitm*Del−/− MEFs stably overexpressing Ifitm3 (*Ifitm*Del−/−*Ifitm3*), were left untreated (left panels, Buffer), or treated (right panels) with IFN-γ. The following day cells were incubated on ice with PR8 (moi 500). Cells were next incubated in warm media containing LTRed (0 min.). Cells were then fixed at the indicated times p.i., immunostained with anti-NP antibodies (green) and imaged by confocal microscopy. Image analysis software was used to define the nuclear boundaries (blue lines). Images are representative of three independent experiments. (Scale bar 12 γM). B) Percent colocalization of vRNP and LTRed compartments in the indicated MEF cell lines, with or without IFN-γ treatment, are shown for the indicated times p.i. C) Quantitation of nuclear vRNP particles. The number of vRNP particles per nucleus of the MEF cell lines, with or without IFN-γ treatment, at the indicated time points are shown. Values represent the mean +/− the SD of three independent experiments. D) Western blot of whole cell lysates from the indicated MEFs probed with anti-mouse Ifitm3 and using GAPDH as a loading control.
(PDF)

Figure S7 **Fusion of viral pseudoparticles expressing HA envelope subtypes, but not a MLV envelope, is decreased by IFITM3. IFITM3 inhibits the replication of infectious H5N1 virus.** A) MDCK cells stably transduced with IFITM3 or empty vector were incubated with pseudoparticles expressing N1 and HA subtypes (H1N1pp, H3N1pp, or H5N1pp). Cells were then fixed and assayed for cleavage of CCF2 using flow cytometry. These results are representative of three independent experiments. B) Chicken embryonic fibroblasts (ChEF) cells stably expressing the empty vector control or IFITM3 were incubated with pseudoparticles expressing N1 and either of the two avian influenza A viral HA subtypes, H5 or H7, as in (A). These data are representative of three independent experiments. C) ChEF cells stably transduced with the empty vector control or overexpressing IFITM3, were infected with WSN/33 for 12 h then stained for HA protein (red) and DNA (blue). Average percent infection is given for three independent experiments +/− SD. 4× magnification. D) MDCK-Vector or MDCK-IFITM3 cells were incubated with pseudoparticles expressing the amphotropic MLV envelope protein (MLVpp) and then assayed for cleavage of CCF2 using flow cytometry. These results are representative of two independent experiments. E) Infectivity of HA-expressing pseudoparticles is decreased by IFITM3. MDCK-Vector or MDCK-IFITM3 cell lines were infected with the indicated pseudoparticles for 48 h. Cells were then immunostained for expression of HIV-1 p24 protein expressed from the integrated lentiviral genomes. Percent infection is provided. These results are representative of three independent experiments. 4× magnification. F) A549 cells were stably transduced with retroviruses containing IFITM3 or empty viral vector alone, then infected with A/Vietnam/1203/04 (H5N1) influenza A virus (VN/04). After 12 h, the cells were fixed and stained for viral NP expression (green) and for DNA (blue). Values given are percentage infected cells and are representative of two independent experiments. 4× magnification. G) Western blot of lysates from A549-IFITM3 or A549-Vector cell lines probed with the indicated antibodies. H) A549 cell lines were infected with increasing amounts of H5N1 VN/04. Twelve hours after infection the cells were immunostained for NP expression and scored for infection status. Values are representative of two independent experiments.
(PDF)

Figure S8 **IFITM3 is required for IFN's inhibition of HA-mediated fusion.** A) HeLa cells were stably transduced with retroviruses containing either IFITM3, a shRNA against IFITM3 (shIFITM3), or a non-targeting control shRNA (shScr). Cells were left untreated (left panels), or treated with IFN-γ (right panels), then exposed for 2 h to H1N1pps (WSN/33) containing BLAM-Vpr. After incubation with the pseudoparticles, the cells were fixed and assayed for cleavage of CCF2 by flow cytometry. These results are representative of three independent experiments. B) The indicated HeLa cell lines were treated with IFN-γ for 24 h then infected with increasing amounts of WSN/33. After 12 h of infection the cells were stained for HA expression. These results are representative of three independent experiments. C) Western blot of the indicated HeLa cell line lysates probed with the indicated antibodies.
(PDF)

Figure S9 **A549 cells depleted of IFITM3 show increased susceptibility to influenza A virus infection. MxA is expressed and is IFN-inducible in A549 and WI-38 cells.** A) A549 cells, stably transduced with retroviruses expressing IFITM3, a negative control shRNA against firefly luciferase (shLuc), or one of three shRNAs against IFITM3 (1, 2 or 3), were treated with buffer, IFN-α or IFN-γ for 24 h, then challenged with WSN/33. After 12 h of infection, the cells were fixed and immunostained for HA and stained for DNA. IF images were captured and the percentage of infected cells determined based on HA staining. Values represent the average of three independent experiments +/−SD. B) Western lysates of A549 cells from (A) probed with the indicated antibodies. Western lysates of (C) A549 cells or (D) WI-38 cells, treated with buffer, IFN-α or -γ, then probed with the indicated antibodies. E) Confocal images of WI-38 cells treated with buffer or IFN-α, then fixed, permeabilized and immunostained for either IFITM3 (endogenous, red), or MxA (endogenous, green), and for DNA (blue, scale bar: 20 µM).
(PDF)

Figure S10 **IFN treatment both expands Rab7- and IFITM3-containing structures, and increases the size and number of acidified organelles.** A) Confocal images of WI-38 cells treated with buffer, IFN-α, or IFN-γ, and then immunostained for either IFITM3 (endogenous, red) or Rab7 (endogenous, green), and DNA (blue). Arrows denote larger structures staining for Rab7 and IFITM3 that were seen predominantly with IFN-γ treatment. (Scale bar: 20 µM). B) A549 cells treated with either buffer or IFN-γ, then incubated with LTRed before fixation and DNA staining (blue) followed by confocal imaging. Images in this figure are representative of three independent experiments.
(PDF)
