# Supplementary figures and images for: IFITM3 Inhibits Influenza A Virus Infection by Preventing Cytosolic Entry
Source: PLoS Pathog. 2011 Oct 27;7(10):e1002337. doi: 10.1371/journal.ppat.1002337 (PMC3203188; doi:10.1371/journal.ppat.1002337)

**A**

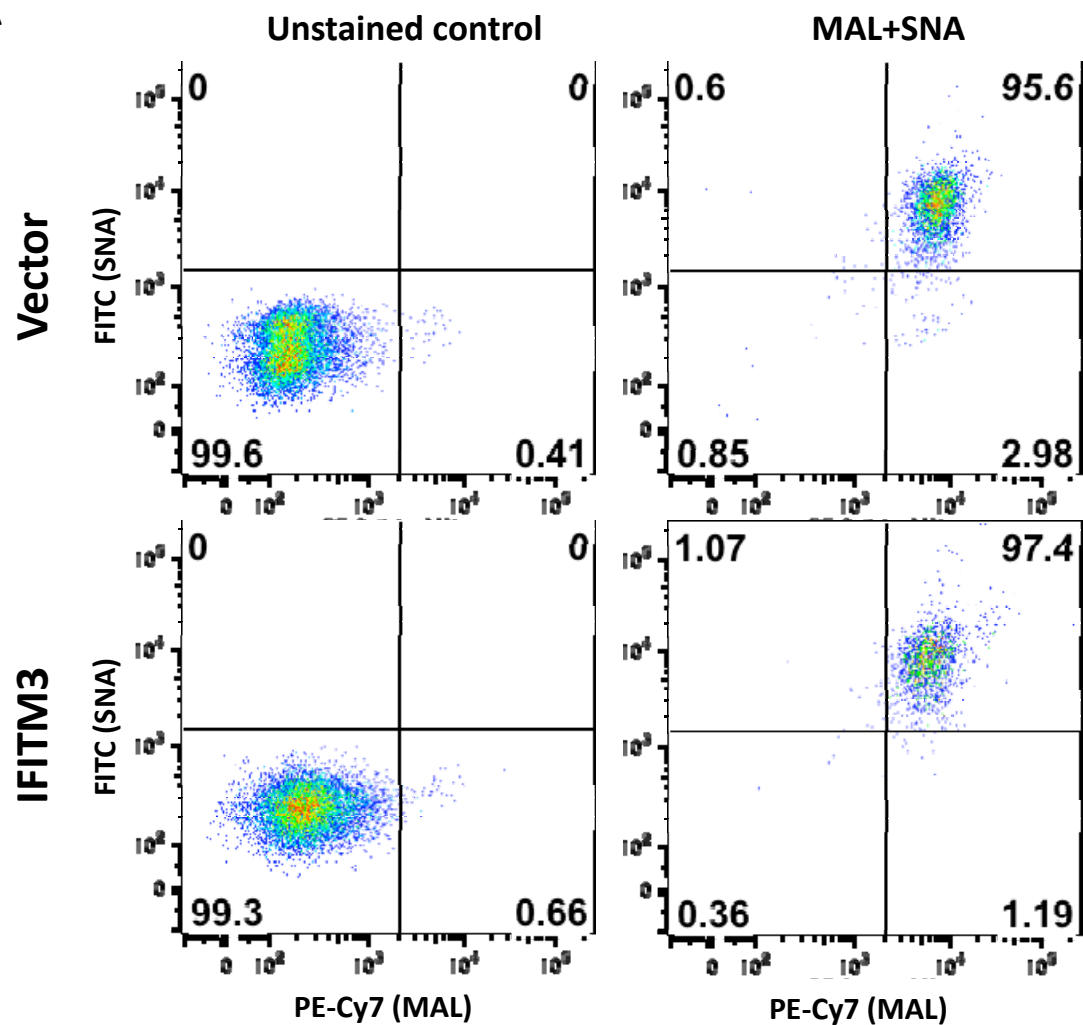

**B**

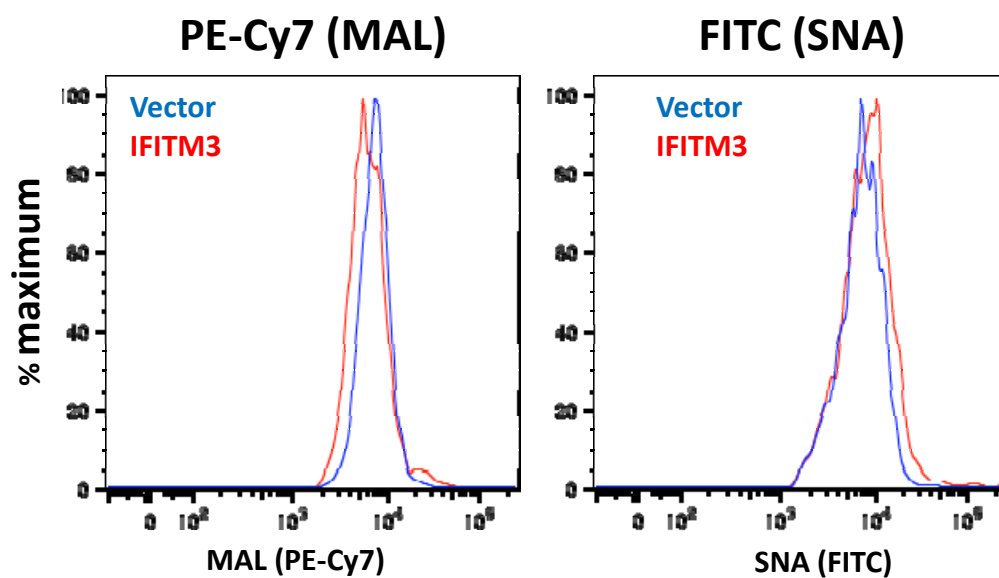

Supplement: Figure S1 — IFITM3 overexpression does not alter the surface levels of (α-2,3) or (α-2,6) sialylated proteins. A549 cells stably transduced with IFITM3 or the empty vector were incubated with biotinylated Maackia Amurensis lectin II (MAL) to detect (α-2,3) sialic acid linkages, followed by streptavidin-PE-Cy7, as well as FITC-conjugated Sambucus Nigra lectin (SNA) to detect (α-2,6) sialic acid linkages. A) The percentage of IFITM3 or vector cells staining positive for both sialic acid linkages (upper right hand quadrant), compared to unstained controls. B) IFITM3 overexpressing and vector cells are compared with regard to each sialic acid linkage in the double-stained populations. (PDF) [file ppat.1002337.s001.pdf]

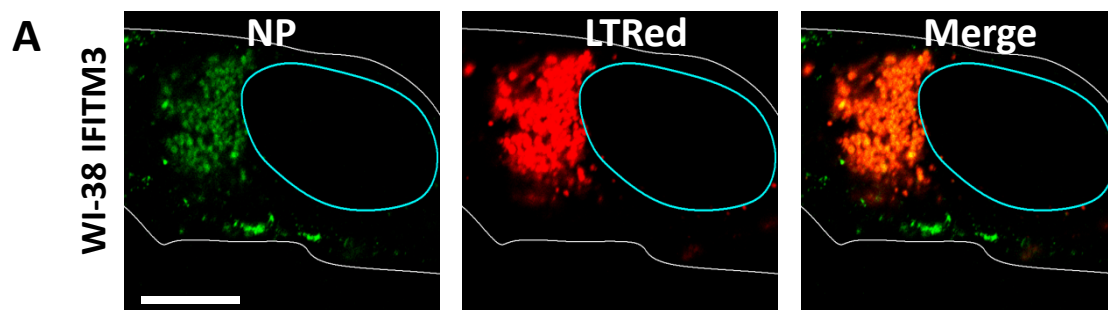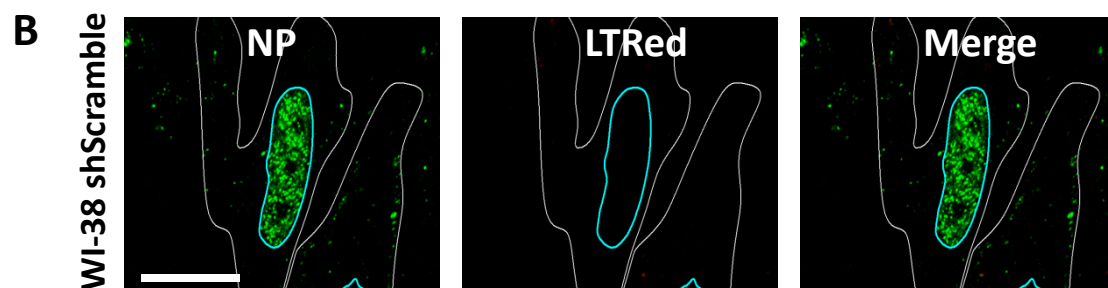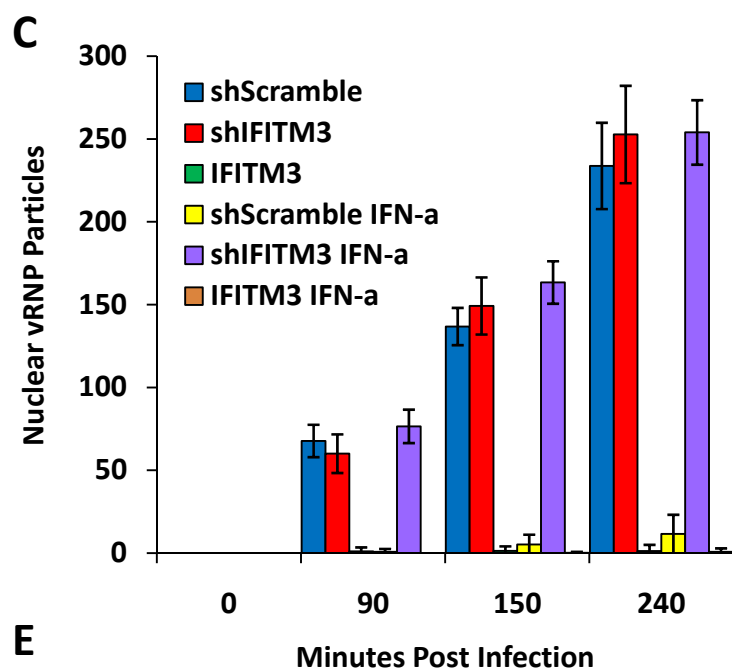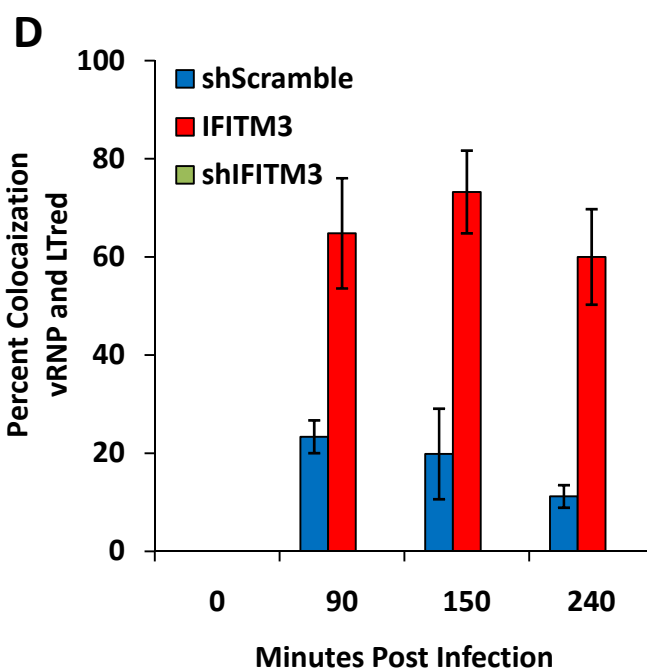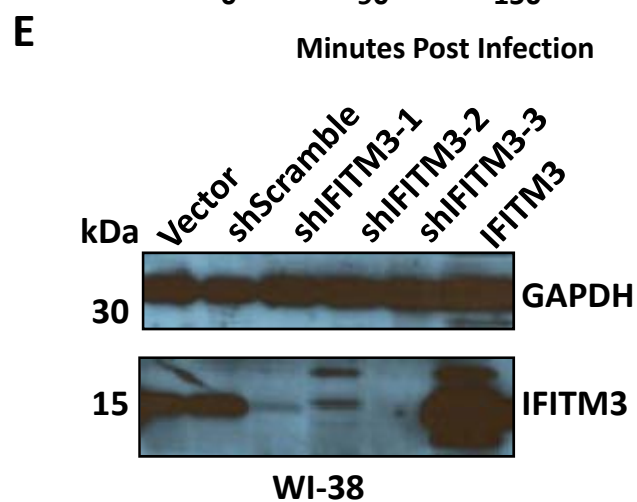

Supplement: Figure S2 — IFITM3 arrests influenza A virus in acidic cytosolic inclusions preventing vRNP nuclear translocation. A) Normal diploid human fibroblasts (WI-38 cells) were stably transduced with retroviruses containing IFITM3 (WI-38 IFITM3) or (B) a non-targeting control shRNA (WI-38 shScramble). Cells were incubated with PR8 on ice, and then warm media containing LTRed (red) was added at time zero. Cells were fixed at 150 min. p.i. and stained for NP (green) and DNA, then analyzed by confocal microscopy. Image analysis software was used to define each cell's cytosolic (white lines) and nuclear peripheries (blue lines, based on DIC images and DNA staining, respectively). Images are representative of four independent experiments. (Scale bar: 12 µM). C) Quantitation of nuclear vRNP particles. The number of vRNP particles per nucleus of the WI-38 cell lines (with or without IFN treatment) at the indicated time points are shown. Values represent the mean +/− the SD of three independent experiments. D) Percent colocalization of vRNPs and LTRed compartments in WI-38 shScramble, shIFITM3 or IFITM3 expressing cells at the indicated times p.i. Values represent the mean +/− the SD of three independent experiments. E) Western blot of lysates from WI-38 cells probed with the indicated antibodies. shIFITM3-3 is referred to as shIFITM3 in the preceding figures and was selected for use based on its superior knockdown of the target protein. (PDF) [file ppat.1002337.s002.pdf]

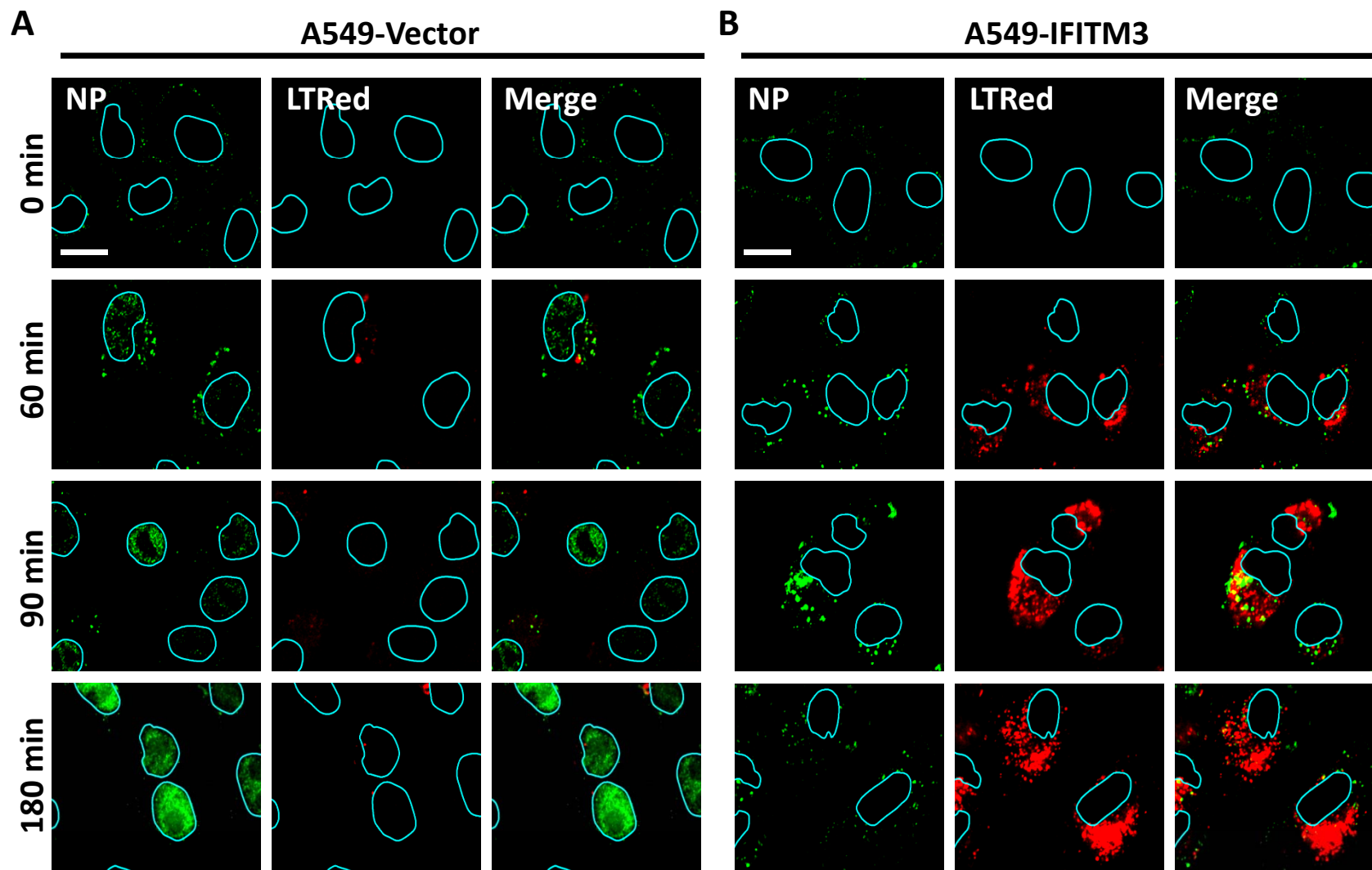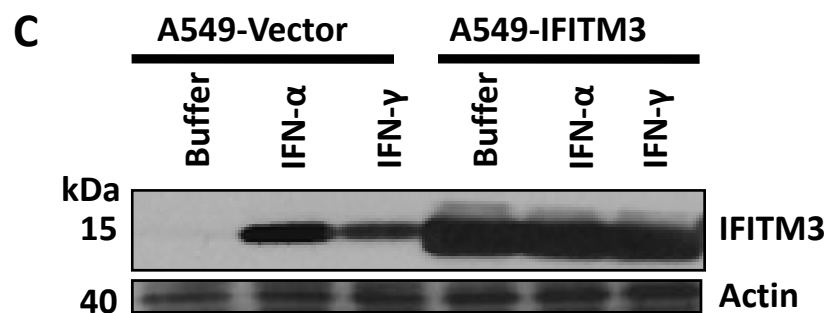

Supplement: Figure S3 — A549 cells overexpressing IFITM3 inhibit vRNP nuclear entry. A549 cells overexpressing the empty vector control (A) or IFITM3 (B) were incubated with PR8 on ice (moi 500). At time zero warm media was added along with LTRed (red). At the indicated times, cells were processed and stained for NP (green) and DNA (blue lines represent the nuclear periphery based on staining), then imaged using a confocal microscope. (Scale bar: 20 µM). These images are representative of three independent experiments. C) Whole cell lysates of A549 cells used in (A) and (B) treated with either buffer, IFN-α or IFN-γ, were subjected to immunoblotting using the indicated antibodies. (PDF) [file ppat.1002337.s003.pdf]

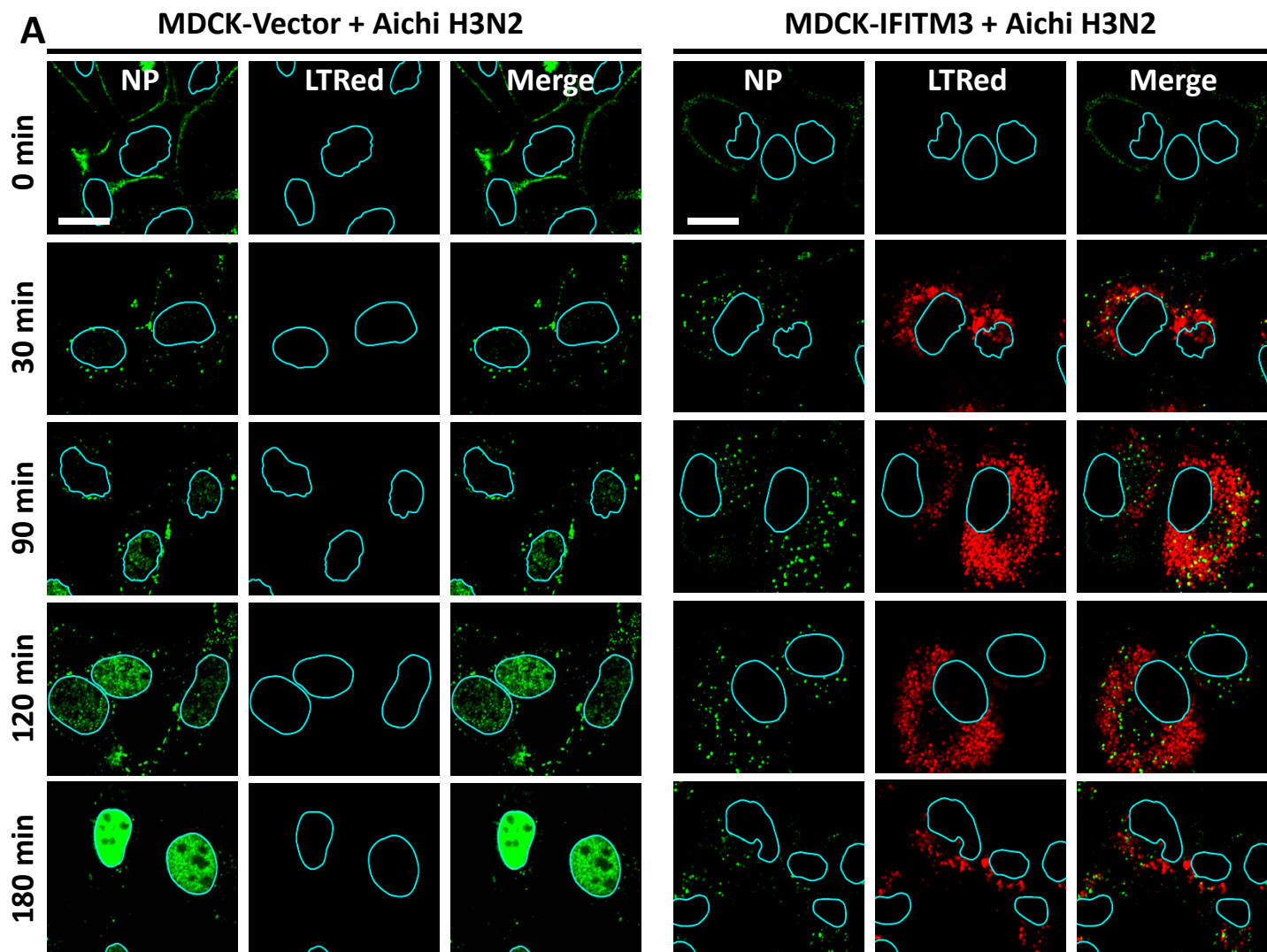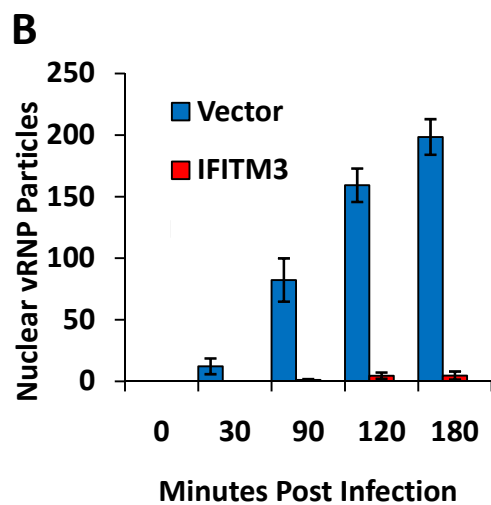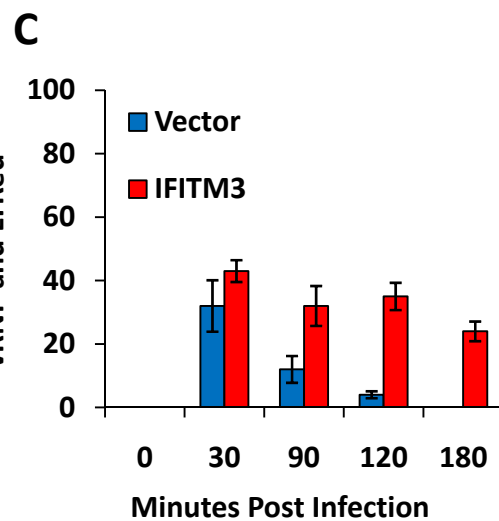

Supplement: Figure S4 — IFITM3 overexpression halts H3N2 influenza A virus in acidic cytosolic inclusions prior to vRNP nuclear translocation. MDCK cells stably expressing (A) the empty vector control or (B) IFITM3 were incubated with Aichi H3N2 virus on ice, and then warm media was added at time zero along with LTRed (red). Cells were then fixed at the indicated times p.i. and stained for NP (green), and DNA (blue lines denote nuclear periphery), then imaged by confocal microscopy. Images are representative of three independent experiments. (Scale bar: 20 µm). B) Quantitation of nuclear vRNP particles. The number of vRNP particles per nucleus of the MDCK cell lines at the indicated time points are shown. Values represent the mean +/− the SD of three independent experiments. C) Percent colocalization of vRNP and LTRed compartments in MDCK-Vector and MDCK-IFITM3 cell lines at the indicated times p.i. (PDF) [file ppat.1002337.s004.pdf]

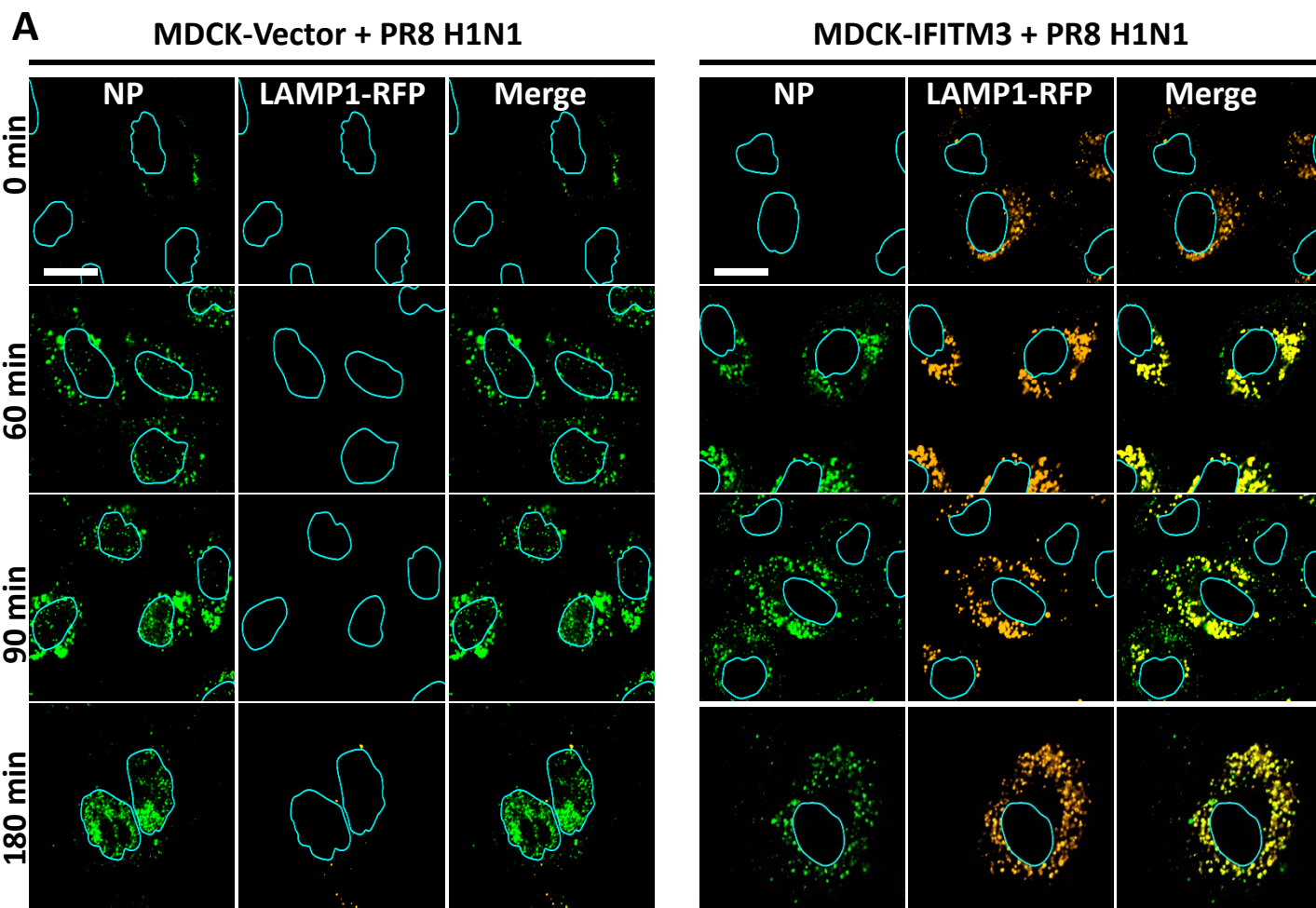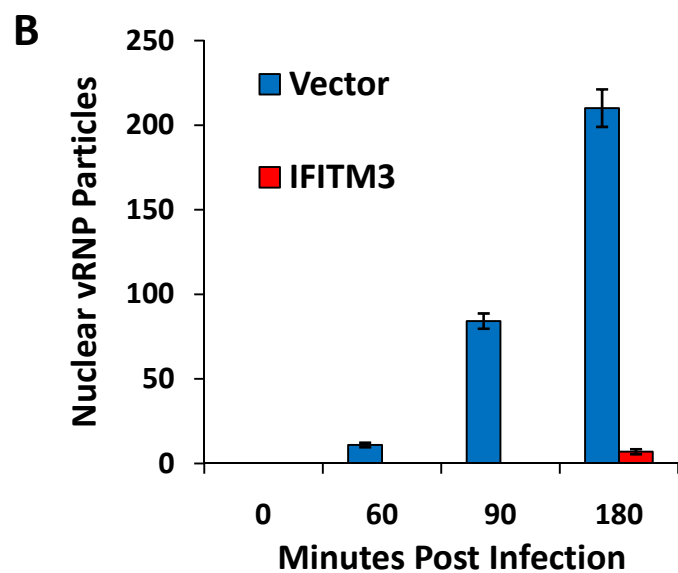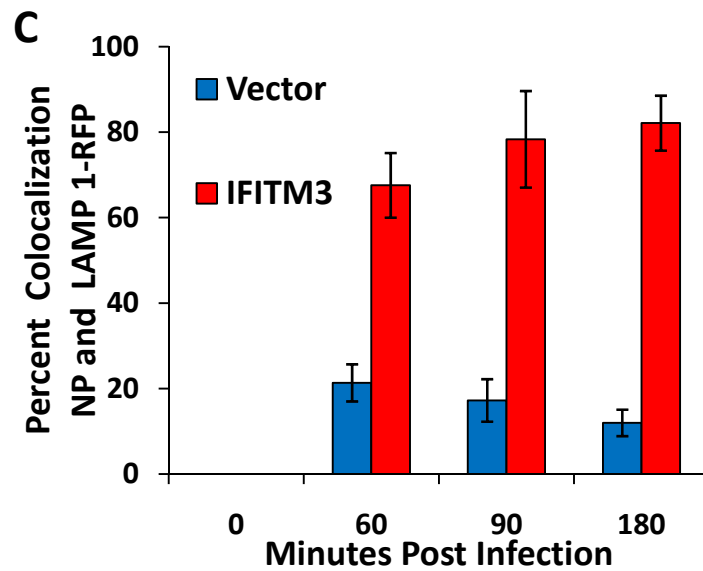

Supplement: Figure S5 — vRNPs are retained in LAMP1-containing organelles in cells overexpressing IFITM3. A) MDCK-Vector or IFITM3 cells stably expressing a LAMP1-red fluorescence protein (LAMP1-RFP) were challenged with PR8 as in Fig. S4. Cells were immunostained for NP (green), stained for DNA, and then imaged confocally along with the collection of LAMP1-RFP fluorescence (orange). Images are representative of three independent experiments. Blue lines represent the nuclear margins based on DNA staining. (Scale bar: 20 µM). B) Quantitation of nuclear vRNP particles. The number of vRNP particles present per nucleus of the MDCK cell lines at the indicated time points are shown. Values represent the mean +/− the SD of three independent experiments. C) Percent colocalization of vRNP particles and LAMP1-RFP-containing compartments in MDCK-Vector and MDCK-IFITM3 cell lines at the indicated times p.i. Values represent the mean +/− the SD of three independent experiments. (PDF) [file ppat.1002337.s005.pdf]

# **A** *IfitmDel* $-/-$ *Ifitm3* Buffer

# *IfitmDel* $-/-$ *Ifitm3* IFN- $\gamma$

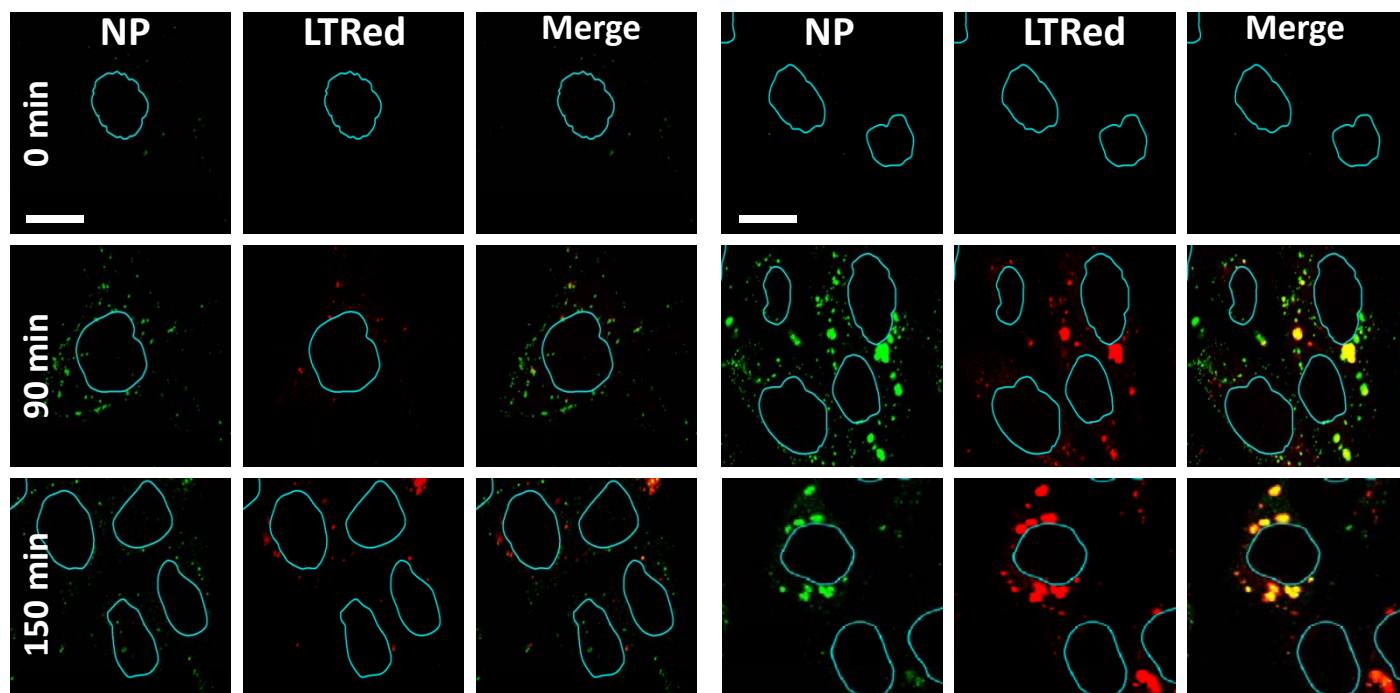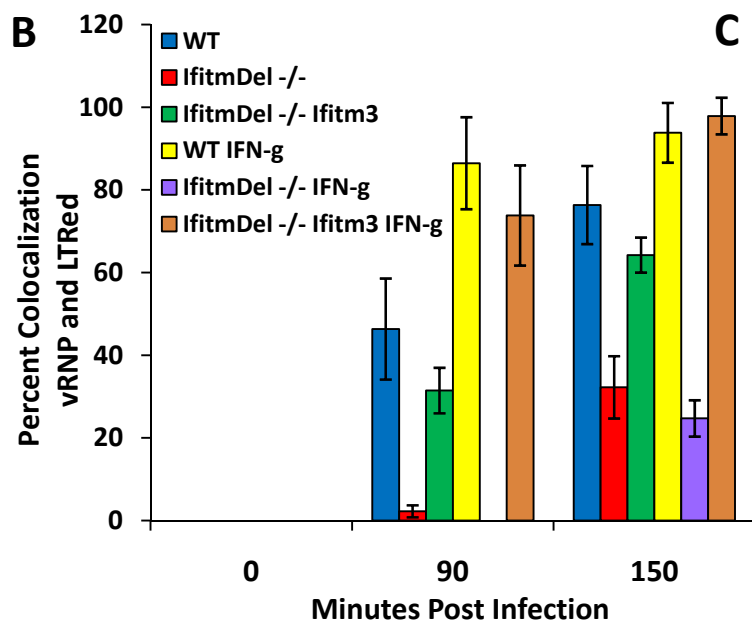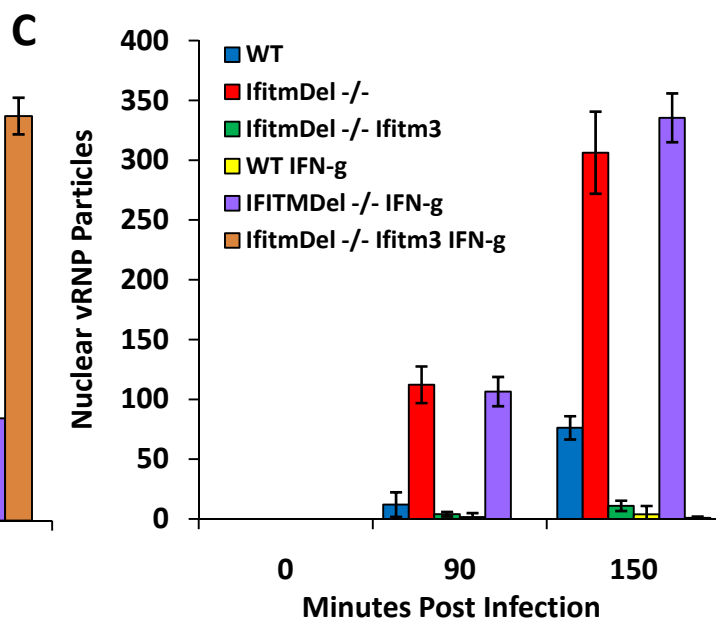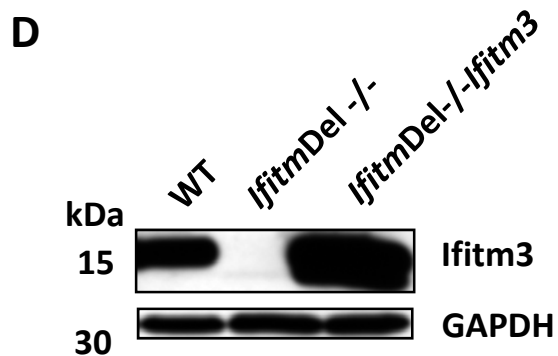

Supplement: Figure S6 — Ifitm3 expression rescues IFN-γ-mediated inhibition of vRNP nuclear translocation in Ifitm Del −/− MEFs. A) IfitmDel−/− MEFs stably overexpressing Ifitm3 (IfitmDel−/−Ifitm3), were left untreated (left panels, Buffer), or treated (right panels) with IFN-γ. The following day cells were incubated on ice with PR8 (moi 500). Cells were next incubated in warm media containing LTRed (0 min.). Cells were then fixed at the indicated times p.i., immunostained with anti-NP antibodies (green) and imaged by confocal microscopy. Image analysis software was used to define the nuclear boundaries (blue lines). Images are representative of three independent experiments. (Scale bar 12 γM). B) Percent colocalization of vRNP and LTRed compartments in the indicated MEF cell lines, with or without IFN-γ treatment, are shown for the indicated times p.i. C) Quantitation of nuclear vRNP particles. The number of vRNP particles per nucleus of the MEF cell lines, with or without IFN-γ treatment, at the indicated time points are shown. Values represent the mean +/− the SD of three independent experiments. D) Western blot of whole cell lysates from the indicated MEFs probed with anti-mouse Ifitm3 and using GAPDH as a loading control. (PDF) [file ppat.1002337.s006.pdf]

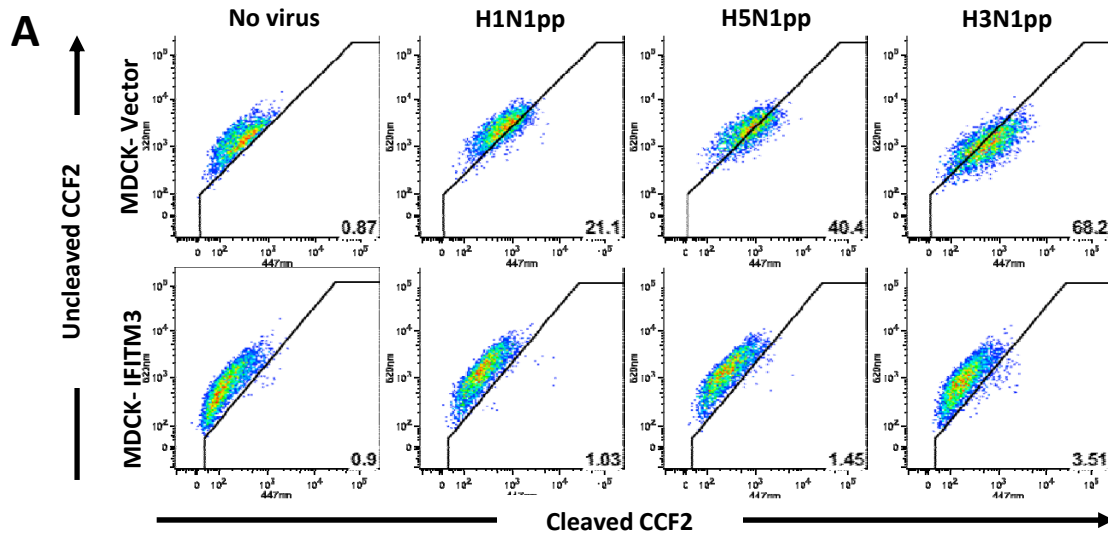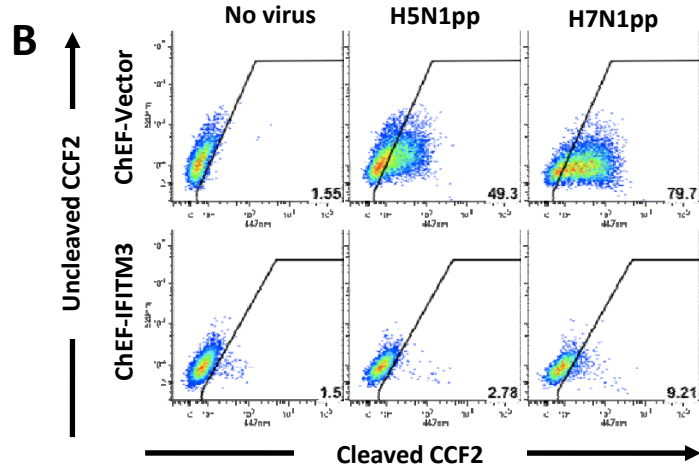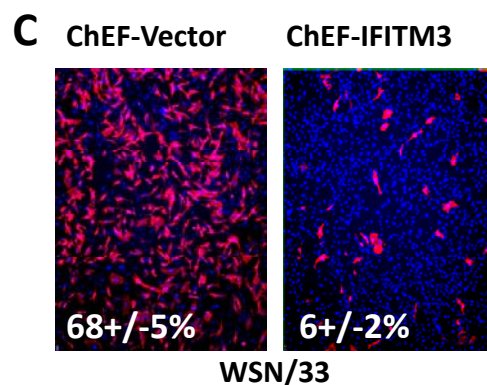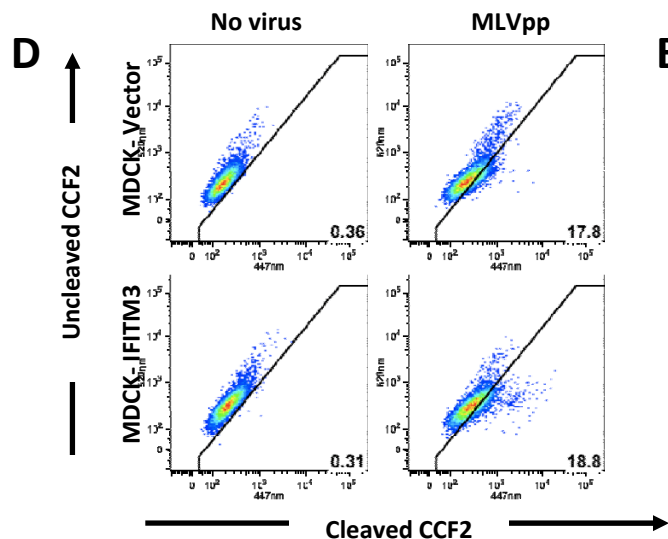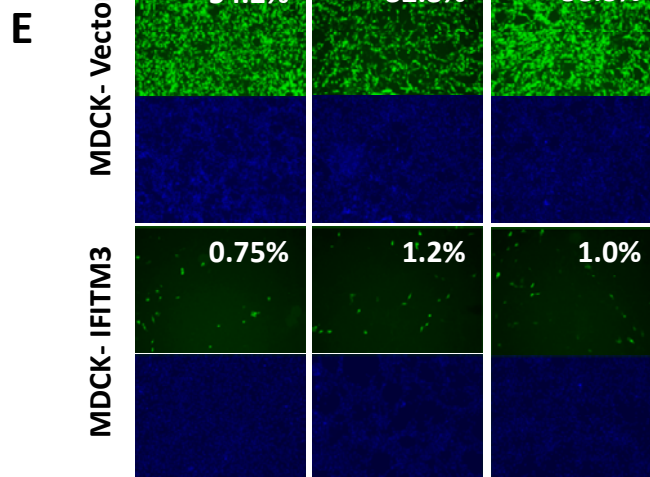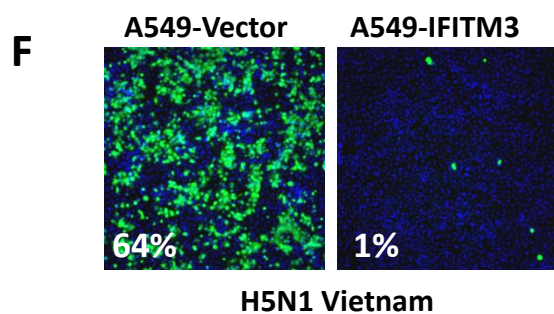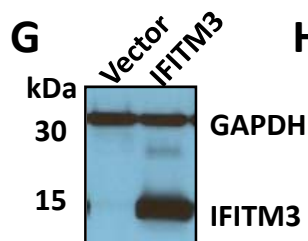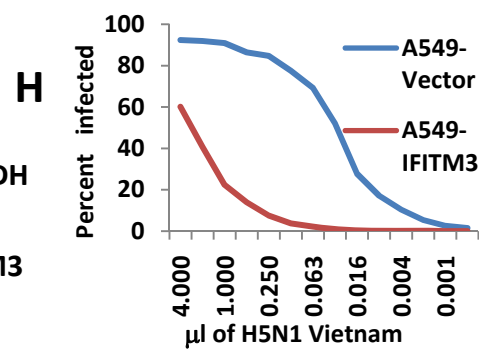

Supplement: Figure S7 — Fusion of viral pseudoparticles expressing HA envelope subtypes, but not a MLV envelope, is decreased by IFITM3. IFITM3 inhibits the replication of infectious H5N1 virus. A) MDCK cells stably transduced with IFITM3 or empty vector were incubated with pseudoparticles expressing N1 and HA subtypes (H1N1pp, H3N1pp, or H5N1pp). Cells were then fixed and assayed for cleavage of CCF2 using flow cytometry. These results are representative of three independent experiments. B) Chicken embryonic fibroblasts (ChEF) cells stably expressing the empty vector control or IFITM3 were incubated with pseudoparticles expressing N1 and either of the two avian influenza A viral HA subtypes, H5 or H7, as in (A). These data are representative of three independent experiments. C) ChEF cells stably transduced with the empty vector control or overexpressing IFITM3, were infected with WSN/33 for 12 h then stained for HA protein (red) and DNA (blue). Average percent infection is given for three independent experiments +/− SD. 4× magnification. D) MDCK-Vector or MDCK-IFITM3 cells were incubated with pseudoparticles expressing the amphotropic MLV envelope protein (MLVpp) and then assayed for cleavage of CCF2 using flow cytometry. These results are representative of two independent experiments. E) Infectivity of HA-expressing pseudoparticles is decreased by IFITM3. MDCK-Vector or MDCK-IFITM3 cell lines were infected with the indicated pseudoparticles for 48 h. Cells were then immunostained for expression of HIV-1 p24 protein expressed from the integrated lentiviral genomes. Percent infection is provided. These results are representative of three independent experiments. 4× magnification. F) A549 cells were stably transduced with retroviruses containing IFITM3 or empty viral vector alone, then infected with A/Vietnam/1203/04 (H5N1) influenza A virus (VN/04). After 12 h, the cells were fixed and stained for viral NP expression (green) and for DNA (blue). Values given are percentage infected cells an [file ppat.1002337.s007.pdf]

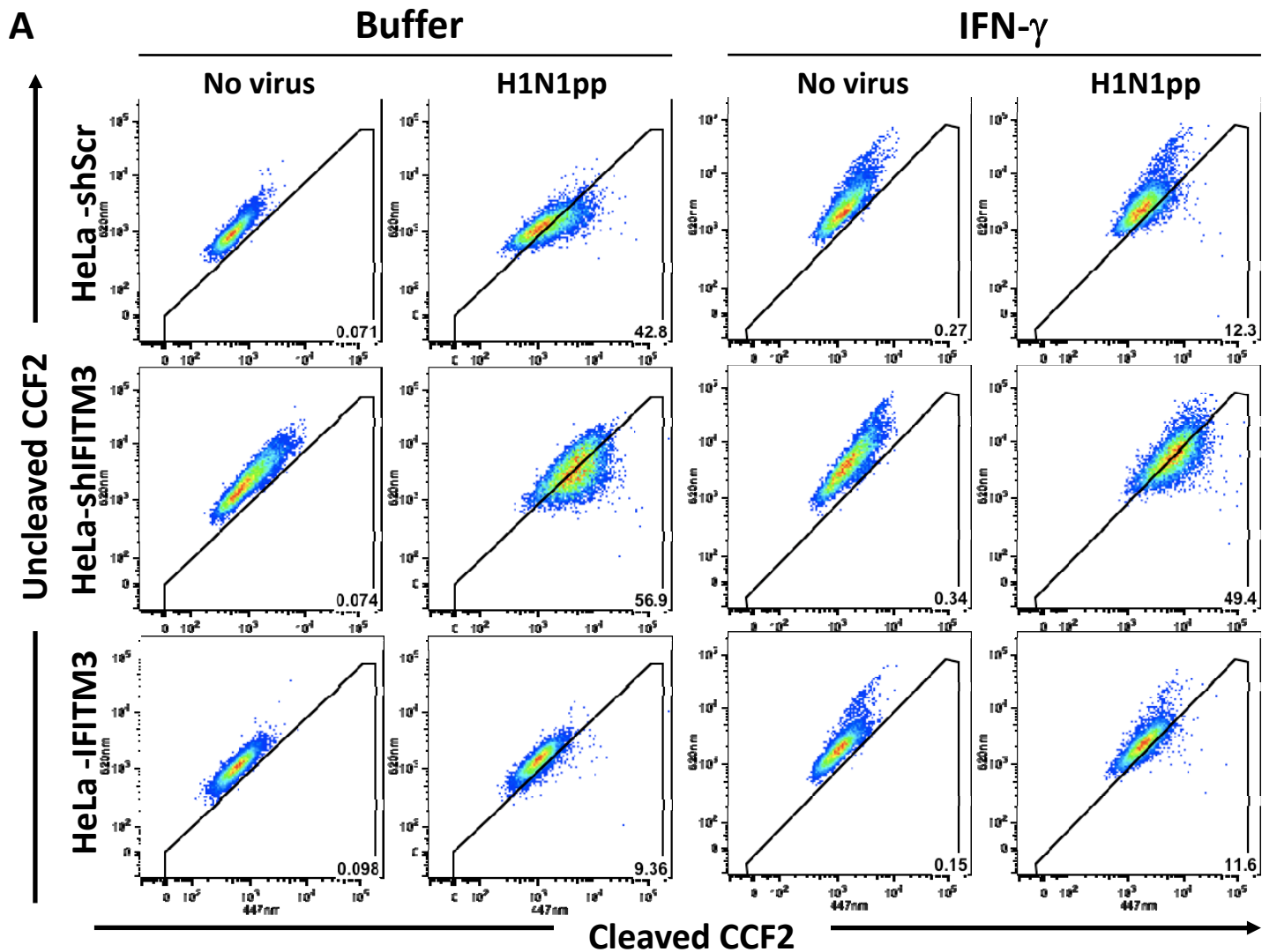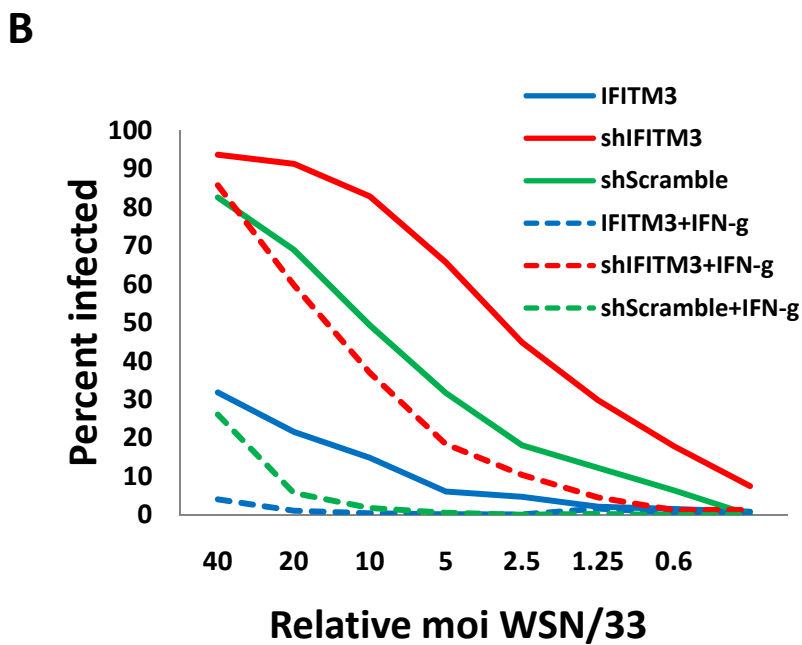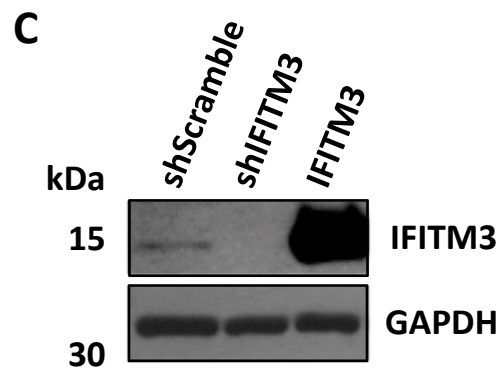

Supplement: Figure S8 — IFITM3 is required for IFN's inhibition of HA-mediated fusion. A) HeLa cells were stably transduced with retroviruses containing either IFITM3, a shRNA against IFITM3 (shIFITM3), or a non-targeting control shRNA (shScr). Cells were left untreated (left panels), or treated with IFN-γ (right panels), then exposed for 2 h to H1N1pps (WSN/33) containing BLAM-Vpr. After incubation with the pseudoparticles, the cells were fixed and assayed for cleavage of CCF2 by flow cytometry. These results are representative of three independent experiments. B) The indicated HeLa cell lines were treated with IFN-γ for 24 h then infected with increasing amounts of WSN/33. After 12 h of infection the cells were stained for HA expression. These results are representative of three independent experiments. C) Western blot of the indicated HeLa cell line lysates probed with the indicated antibodies. (PDF) [file ppat.1002337.s008.pdf]

**A**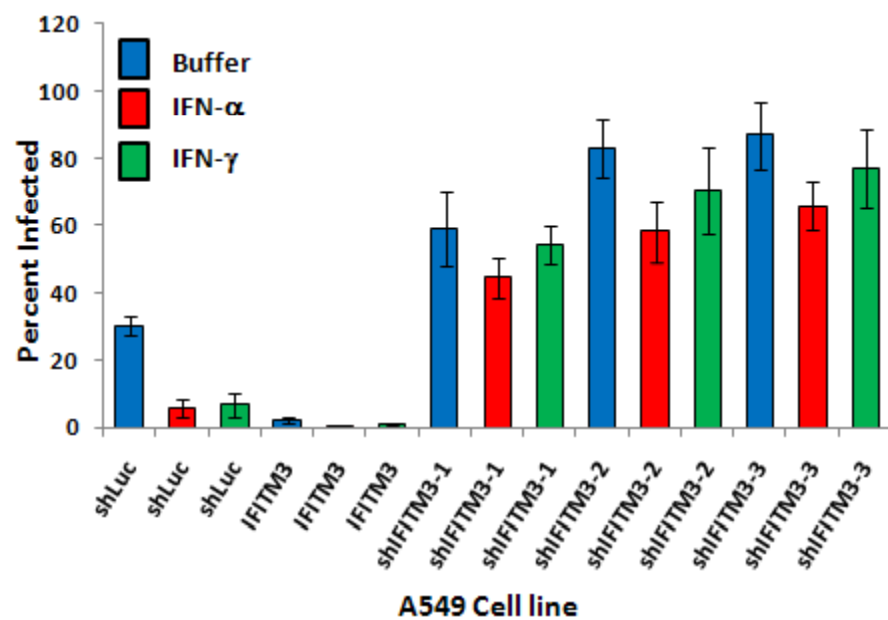**B**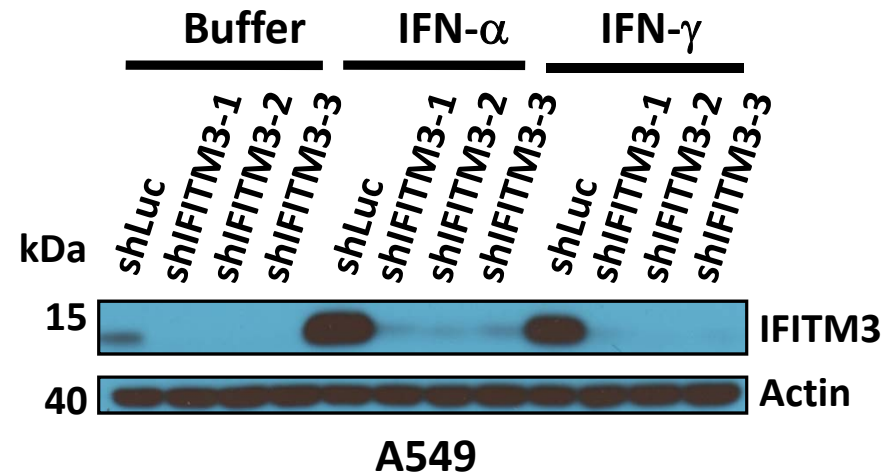**C**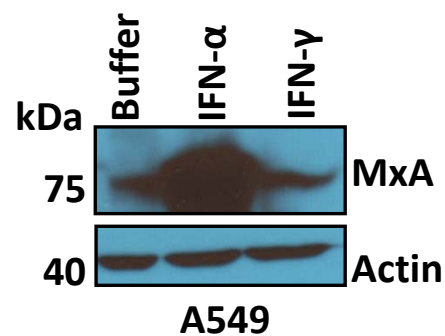**D**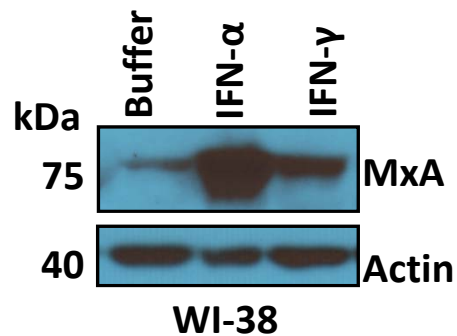**E**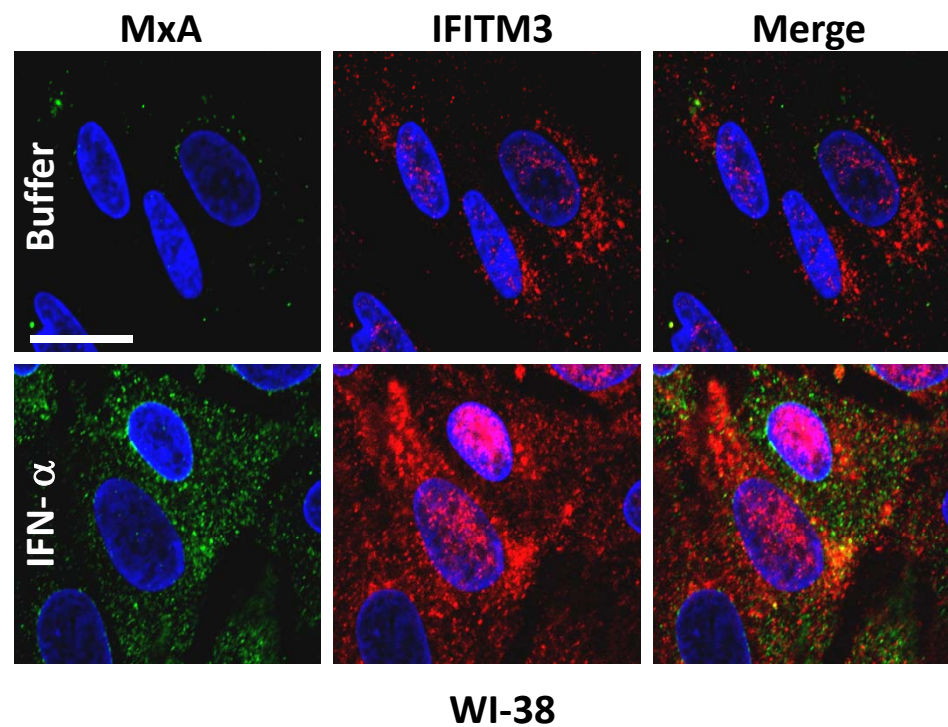

Supplement: Figure S9 — A549 cells depleted of IFITM3 show increased susceptibility to influenza A virus infection. MxA is expressed and is IFN-inducible in A549 and WI-38 cells. A) A549 cells, stably transduced with retroviruses expressing IFITM3, a negative control shRNA against firefly luciferase (shLuc), or one of three shRNAs against IFITM3 (1, 2 or 3), were treated with buffer, IFN-α or IFN-γ for 24 h, then challenged with WSN/33. After 12 h of infection, the cells were fixed and immunostained for HA and stained for DNA. IF images were captured and the percentage of infected cells determined based on HA staining. Values represent the average of three independent experiments +/−SD. B) Western lysates of A549 cells from (A) probed with the indicated antibodies. Western lysates of (C) A549 cells or (D) WI-38 cells, treated with buffer, IFN-α or -γ, then probed with the indicated antibodies. E) Confocal images of WI-38 cells treated with buffer or IFN-α, then fixed, permeabilized and immunostained for either IFITM3 (endogenous, red), or MxA (endogenous, green), and for DNA (blue, scale bar: 20 µM). (PDF) [file ppat.1002337.s009.pdf]

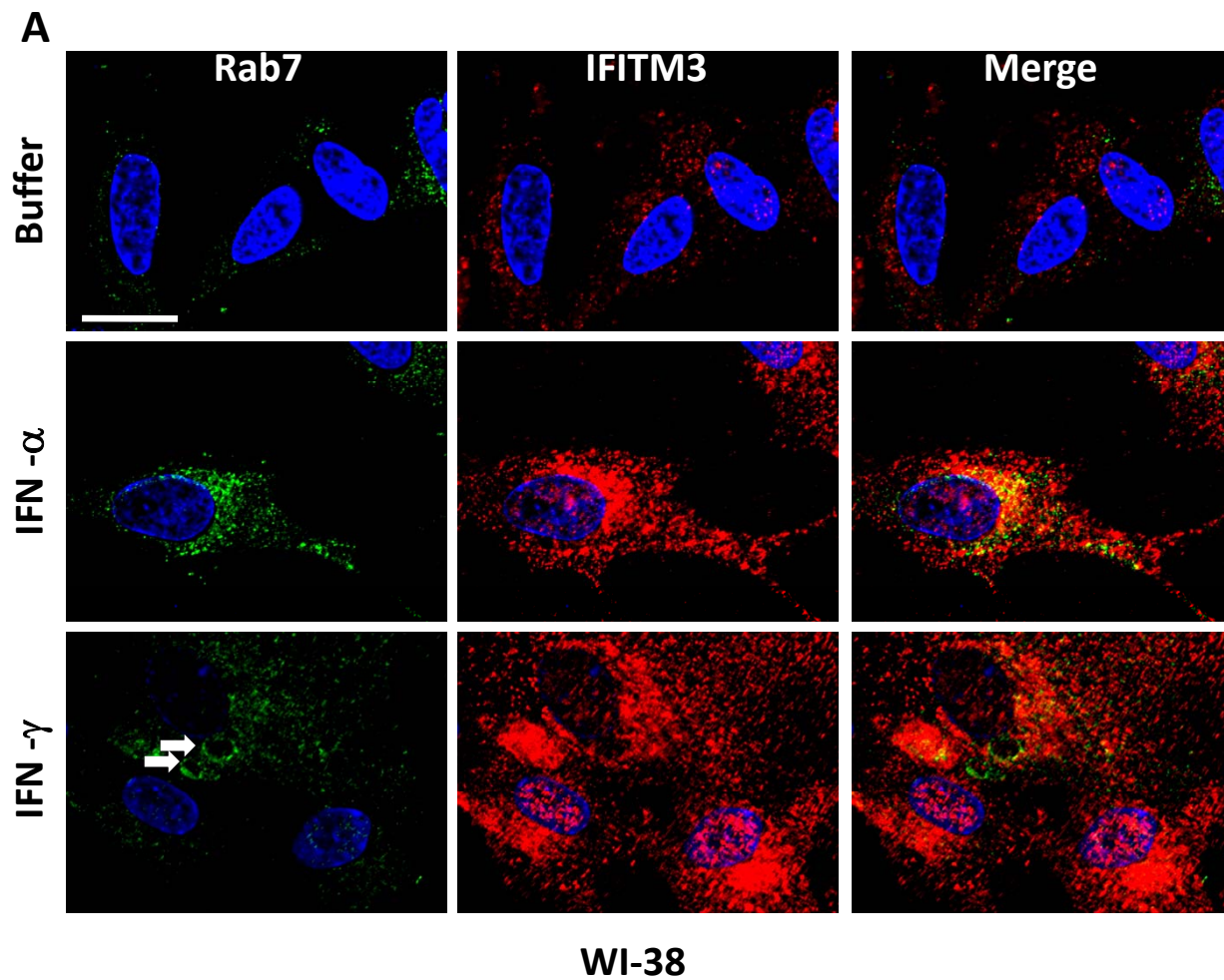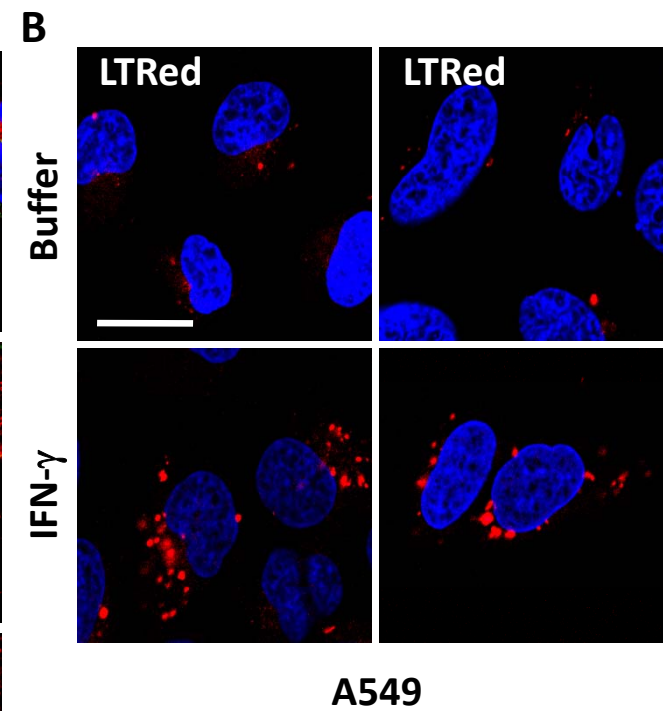

Supplement: Figure S10 — IFN treatment both expands Rab7- and IFITM3-containing structures, and increases the size and number of acidified organelles. A) Confocal images of WI-38 cells treated with buffer, IFN-α, or IFN-γ, and then immunostained for either IFITM3 (endogenous, red) or Rab7 (endogenous, green), and DNA (blue). Arrows denote larger structures staining for Rab7 and IFITM3 that were seen predominantly with IFN-γ treatment. (Scale bar: 20 µM). B) A549 cells treated with either buffer or IFN-γ, then incubated with LTRed before fixation and DNA staining (blue) followed by confocal imaging. Images in this figure are representative of three independent experiments. (PDF) [file ppat.1002337.s010.pdf]
